# Molecular basis of pyruvate transport and inhibition of the human mitochondrial pyruvate carrier

**DOI:** 10.1126/sciadv.adw1489

**Published:** 2025-04-18

**Authors:** Maximilian Sichrovsky, Denis Lacabanne, Jonathan J. Ruprecht, Jessica J. Rana, Klaudia Stanik, Mariangela Dionysopoulou, Alice P. Sowton, Martin S. King, Scott A. Jones, Lee Cooper, Steven W. Hardwick, Giulia Paris, Dimitri Y. Chirgadze, Shujing Ding, Ian M. Fearnley, Shane M. Palmer, Els Pardon, Jan Steyaert, Vanessa Leone, Lucy R. Forrest, Sotiria Tavoulari, Edmund R. S. Kunji

**Affiliations:** ^1^ MRC Mitochondrial Biology Unit, University of Cambridge, Keith Peters Building, Cambridge Biomedical Campus, Cambridge CB2 0XY, UK.; ^2^ Computational Structural Biology Section, National Institutes of Neurological Disorders and Stroke, NIH, Bethesda, MD 20892, USA.; ^3^ Department of Biochemistry, University of Cambridge, Sanger Building, Tennis Court Road, Cambridge CB2 1GA, UK.; ^4^ VIB-VUB Center for Structural Biology, VIB, Pleinlaan 2, B-1050 Brussels, Belgium.; ^5^ Structural Biology Brussels, Vrije Universiteit Brussel, Pleinlaan 2, B-1050 Brussels, Belgium.; ^6^ Department of Biophysics and Data Science Institute, Medical College of Wisconsin, Milwaukee, WI 53226-3548, USA.

## Abstract

The mitochondrial pyruvate carrier transports pyruvate, produced by glycolysis from sugar molecules, into the mitochondrial matrix, as a crucial transport step in eukaryotic energy metabolism. The carrier is a drug target for the treatment of cancers, diabetes mellitus, neurodegeneration, and metabolic dysfunction–associated steatotic liver disease. We have solved the structure of the human MPC1L/MPC2 heterodimer in the inward- and outward-open states by cryo–electron microscopy, revealing its alternating access rocker-switch mechanism. The carrier has a central binding site for pyruvate, which contains an essential lysine and histidine residue, important for its ΔpH-dependent transport mechanism. We have also determined the binding poses of three chemically distinct inhibitor classes, which exploit the same binding site in the outward-open state by mimicking pyruvate interactions and by using aromatic stacking interactions.

## Teaser

The mitochondrial pyruvate carrier transport mechanism is ΔpH driven and is inhibited competitively by distinct compound classes.

## INTRODUCTION

The mitochondrial pyruvate carrier (MPC) is responsible for transporting pyruvate, produced from sugars by glycolysis in the cytosol, into the mitochondrial matrix. This key transport step links cytosolic glycolysis with mitochondrial oxidative phosphorylation, increasing the adenosine 5′-triphosphate (ATP) yield by 15-fold. MPC plays a critical role in several physiological and pathological processes, including energy metabolism, development, neurodegeneration, diabetes mellitus, and cancer (*1*). MPC activity can influence several metabolic pathways, including the tricarboxylic acid cycle, gluconeogenesis, the malate-aspartate shuttle, the urea cycle, and lipid metabolism (*1*). Since MPC activity is important for embryonic development, its mutations are rare but result in severe disease phenotypes, including lactic acidosis, brain dysfunction, and developmental abnormalities (*2*–*4*).

Originally, pyruvate was believed to enter mitochondria via diffusion (*5*, *6*). However, pyruvate transport was shown to follow saturation kinetics, to be sensitive to sulfhydryl reagents (*7*), and to be abolished by small-molecule inhibitors (*8*), supporting the existence of a carrier protein, although its molecular identity was not discovered for several decades. In 2012, the proteins responsible for pyruvate transport in the mitochondria of yeast, flies, and humans were identified, showing that MPC consists of two small homologous membrane proteins that form heterocomplexes (*2*, *9*). In the yeast *Saccharomyces cerevisiae*, the Mpc1p/Mpc2p heterocomplex forms predominantly in fermentative conditions, whereas the Mpc1p/Mpc3p heterocomplex forms in aerobic conditions (*10*). Import of the yeast MPC proteins follows the mitochondrial carrier pathway, using the receptor Tom70, small translocase of the inner membrane (TIM) chaperones, and the TIM22 complex (*11*). There are three MPC proteins in humans: MPC1, MPC2, and MPC1L, which is 64% identical to MPC1 (fig. S1). Two alternate heterocomplexes are formed: MPC1/MPC2, ubiquitously expressed in all tissues, and MPC1L/MPC2, which is solely expressed in the testes of placental mammals (*12*). Recently, the reconstitution of yeast and human MPC demonstrated that the functional unit is a heterodimer in both species (*13*, *14*). Topology studies for yeast Mpc1/Mpc3 suggested that Mpc3 had three helices with the N terminus located in the mitochondrial matrix and the C terminus in the intermembrane space, whereas Mpc1 had only two helices with both termini in the matrix (*10*). The same topology was suggested for human MPC2 and MPC1/MPC1L, respectively (*12*). Pyruvate transport by purified MPC reconstituted into liposomes (*13*) and by mitochondria (*15*) depends on a difference in pH across the membrane, but the molecular basis of the ΔpH-dependent transport mechanism remains unknown.

MPC is being assessed as a pharmacological target for different pathologies, including metabolic dysfunction–associated steatohepatitis (MASH; previously known as NASH) (*16*–*18*), type 2 diabetes mellitus (*19*), neurodegenerative disorders (*20*, *21*), and specific cancers (*22*, *23*). Alpha-cyano-cinnamate and derivatives were the first inhibitors developed for MPC (*8*, *24*, *25*). The canonical MPC inhibitor, UK5099, which has a half-maximal inhibitory concentration (IC_50_) of 50 nM, belongs to this group (*24*). A novel derivative, compound 7 (C7), was recently identified with a 10-fold higher potency (IC_50_ of 5 nM) (*14*). Among the MPC inhibitors are several molecules with other primary targets, such as the anticancer agent lonidamine (*14*, *26*, *27*), the peroxisome proliferator-activated receptor–γ (PPARγ) agonists thiazolidinediones (TZDs) (*14*, *28*) and the antibiotic nitrofurantoin (*14*), which have inhibitory potencies in the low micromolar range (*14*). In addition, zaprinast, a guanosine 3′,5′-monophosphate–specific phosphodiesterase inhibitor, and entacapone, used as a combination therapy for Parkinson’s disease, inhibit MPC with mid-nanomolar potencies (*14*). Despite the active research in the development of high-affinity MPC inhibitors (*14*, *19*, *29*, *30*), the molecular properties of inhibitor binding have not been defined.

Here, we present the structure of the human pyruvate carrier complex MPC1L/MPC2 in outward- and inward-open states solved by cryo–electron microscopy (cryo-EM), demonstrating that MPC has an alternating access rocker-switch mechanism. Additionally, we determine the orientation of MPC in native membranes and perform transport, binding, and docking studies to identify the pyruvate and proton binding sites. We analyze the conformational space of MPC1/MPC2 and MPC1L/MPC2 with molecular modeling, showing that MPC moves between the outward- and inward-open states via an occluded state. We also solve the structures of MPC in inhibited states with three inhibitor classes, showing that they all exploit the same binding site, although all of the chemical groups are different. The molecular details reveal the pyruvate and inhibitor binding sites as well as the key elements of the ΔpH-dependent transport mechanism of pyruvate.

## RESULTS

### Orientation of the MPC in the mitochondrial inner membrane

The helical topology and orientation of MPC in the inner membrane are still under debate (*1*, *9*, *10*, *12*). Thus, we have revisited the orientation of the MPC complex in the inner membrane of bovine cardiac mitochondria (*31*). We have isolated submitochondrial particles (SMPs) that are 98.5% inside out on the basis of NADH (reduced nicotinamide adenine dinucleotide):APAD^+^ (3-acetylpyridine adenine dinucleotide) oxidoreductase activity of complex I (*31*). Using limited proteolysis with proteinase K, the C-terminal epitopes of both MPC1 and MPC2 are cleaved in inside-out SMPs in a dose-dependent manner. In right-side-out mitoplasts, we detect an additional fragment of MPC1 and MPC2 ~ 3 kDa lighter, corresponding to cleavage in the N-terminal region, leaving the C-terminal epitope protected (fig. S2, A to D). As a control, the oligomycin-sensitivity conferral protein (OSCP) subunit of ATP synthase in the mitochondrial matrix is protected in mitoplasts but degraded in SMPs, confirming the orientation of the samples (fig. S2A). Furthermore, specific chicken antibodies (fig. S2E) were used to confirm that the C-terminal regions of MPC1L and MPC2 were directly accessible in inside-out SMPs, showing a shift of the entire population in flow cytometry, represented as contour plots (fig. S2, F and G). Thus, these results are consistent with an orientation in which the N-terminal region is located in the intermembrane space and the C-terminal region is in the mitochondrial matrix.

### Structure of the human MPC in the outward-open state

We have solved the structure of the MPC1L/MPC2 heterodimer, which can be purified from yeast mitochondria, preserving all known transport and inhibitory properties (*14*). To increase the size of the complex, we used a Pro-macrobody (PMb) generated by fusing an MPC2-specific nanobody (fig. S3A) to maltose-binding protein (MBP) via a rigid Pro-Pro linker (fig. S3B) (*32*). This complex was large enough for single-particle cryo-EM analysis (fig. S4), and the inhibitor mitoglitazone was used to fix this state. The gold standard Fourier shell correlation (FSC) resolution was 3.79 Å (table S1), but most of the MPC complex was 3.2 to 3.4 Å resolution (fig. S5A), which was sufficient to build an atomic model (fig. S6).

**Fig. 1.**
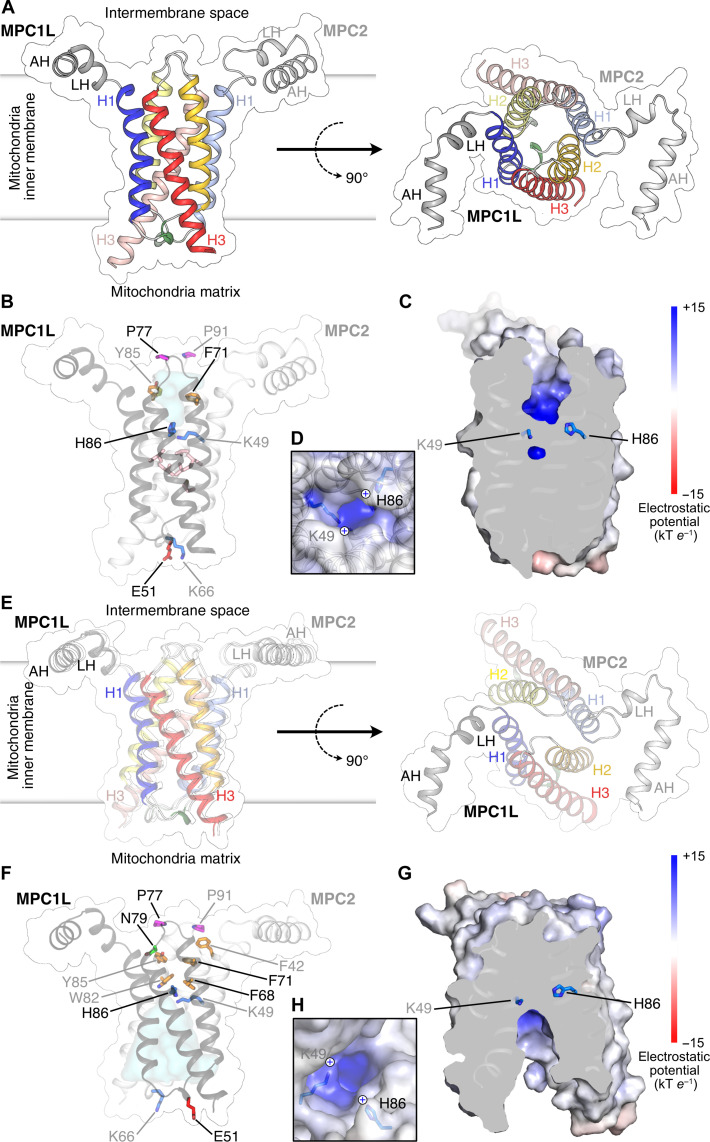
Human MPC in the outward-open and inward-open states. (**A** to **C**) MPC in the mitoglitazone-bound outward-open state. (A) Lateral (left) and cytoplasmic views (right) of the structure. Amphipathic helices (AH) and linker helices (LH) are colored gray, transmembrane helix 1 (H1) is colored blue, helix 2 (H2) is colored yellow, helix 3 (H3) is colored red, and the 3_10_ helix is colored green. The surface is indicated. Lighter colors and labels are used for MPC2. (B) Key residues are shown in stick representation, colored by the Zappo scheme and labeled in black (MPC1L) or gray (MPC2). Residues L38, M57, and L61 from MPC1 and L52, L75, and V103 from MPC2 are shown in pink but are not labeled. The water-accessible cavity is shown in cyan. (C) Cross section through MPC showing the central cavity and (**D**) close-up of the cavity, viewed from the intermembrane space. The surface is colored by electrostatic potential, as shown in the color ramp. K49 and H86 are in stick representation. The view is rotated compared to (B). (**E** to **H**) MPC in the apo inward-open state. (E) Lateral (left) and cytoplasmic views (right). Color scheme as in (A). (F) Key residues and water-accessible cavity, shown as in (B). (G) Central cavity and (H) close-up of the cavity, viewed from the matrix, as in (C) and (D).

Figure 1A shows an overview of the MPC structure in the outward-open state, where the central cavity is open to the intermembrane space. The density map shows that human MPC is a heterodimer (*14*), in which both protomers have an amphipathic helix, a linker helix, and three transmembrane helices joined by short loops. The loop between transmembrane helices H1 and H2 contains a short turn of 3_10_ helix (Fig. 1A). Transmembrane helix H3 is sandwiched between H1 and H2 and is longer than the others, extending beyond the lipid bilayer on the matrix side. The transmembrane helices are arranged around a central twofold pseudosymmetry axis, which lies perpendicular to the membrane plane. The transmembrane helices of both protomers, which form the pyruvate transport domain, are very similar in structure [1.1-Å backbone root mean square deviation (RMSD)] (fig. S7A). Consequently, MPC has a barrel-shaped structure with a narrow central water-filled cavity open to the mitochondrial intermembrane space (Fig. 1B). The N-terminal regions of both proteins form long amphipathic helices and short linker helices, joined to transmembrane helices H1. The amphipathic and linker helices have a hydrophobic and hydrophilic side, and they are likely to lie on the surface of the inner membrane (fig. S8). The amphipathic helices break the twofold pseudosymmetry, as they run approximately parallel to one another but with the N termini pointing in the same direction (Fig. 1A). The reason is that the amphipathic and linker helix of MPC2 form a helix-turn-helix through an interaction network between residues D18, E21, N33, and R39 (fig. S8A).

At the intermembrane space, the water-filled central cavity has a radius of ~4 Å, progressively narrowing toward the center of the membrane (Fig. 1B). The cavity is surrounded by amino acids from all three transmembrane helices of both subunits. At the bottom of the cavity are two positively charged residues, H86 from MPC1L and K49 from MPC2, which contribute to a positive electrostatic potential (Fig. 1, C and D). Beneath this layer, a cluster of hydrophobic and aromatic amino acids form a 9-Å-thick hydrophobic barrier that blocks access to the matrix side (Fig. 1, B and C). On this side, the 3_10_ helices from MPC1L and MPC2 are positioned close together (Fig. 1B). On the intermembrane space side, the loops between H2 and H3 on both subunits contain two proline residues, P77 (MPC1L) and P91 (MPC2) in an unusual cis configuration (Fig. 1B). MPC1L and MPC2 interact via an extensive series of hydrophobic contacts over an interface area of 1330 Å^2^, and there are also two inter-subunit salt bridges and four hydrogen bonds (fig. S9A).

To understand the positional differences in resolution, we aligned five AlphaFold 2 models of the same transport state (fig. S10). Although the structure of the transport domain of MPC is nearly identical, there is a lot of positional variation in the N-terminal amphipathic helix of MPC1L and to a lesser extent that of MPC2. The reason for this flexibility is that there are no interactions between the amphipathic helices and the transport domain, other than via the linker helix. The positional variation (fig. S10) correlates well with resolution (fig. S5A), showing relatively high resolution for the transport domain, which degrades sharply toward the N and C termini to the point that they cannot be modeled. Thus, the relatively modest FSC is due to naturally dynamic and flexible parts of MPC, and an increase in the number of processed particles does not lead to a substantial increase in resolution (fig. S11).

### Structure of uninhibited MPC in the inward-open state

When the human MPC/PMb complex was purified in the absence of inhibitors and imaged by cryo-EM (figs. S12 and S13 and table S1), the analysis showed that MPC is in the inward-open conformation, where the central cavity is open to the mitochondrial matrix (Fig. 1E). The resolution determined by gold-standard FSC was 3.65 Å but, for most of the transport domain, was in the region of 3.2 to 3.4 Å (fig. S5B) and lower for the amphipathic helices and C-termini, which are naturally flexible. In this state, E51 (MPC1L) and K66 (MPC2), which form a salt bridge in the outward-open state (6.3-Å distance between Cα atoms), lie on either side of the cavity opening at a distance of ~23 Å (Fig. 1F). The central cavity extends approximately halfway to the middle of the membrane, narrowing toward the center, where residues H86 (MPC1L) and K49 (MPC2) form the roof of the cavity and contribute to a patch of positive electrostatic potential (Fig. 1, G and H). All three transmembrane helices of both subunits contribute residues that line the cavity. On the matrix side, significant gaps are apparent between the C-terminal end of H1 of one subunit and the N-terminal end of H2 of the other subunit. On the intermembrane space side, MPC1L and MPC2 are closely packed by a cluster of bulky aromatic and hydrophobic residues forming a gate, blocking access to the central cavity (Fig. 1, F and G).

Again, the transmembrane helices of the two protomers have very similar structures (fig. S9B). Inter-subunit interactions are provided principally by hydrophobic contacts between multiple residues, contributing to an interface area of 970 Å^2^ (fig. S9B). Three inter-subunit hydrogen bonds involve residues in the loops between H2 and H3 of both subunits, which lie close together in the inward-open state (fig. S9B). Proline residues, P77 (MPC1L) and P91 (MPC2), on the same loop maintain a cis configuration (Fig. 1, B and F).

In both the outward-open and inward-open states, the transmembrane helices of the protomers have very similar positions and are symmetrically arranged around a twofold pseudosymmetrical axis (Fig. 1 and fig. S7, A and B). In agreement, both protomers have a large number of residues that are symmetrically conserved in agreement with them being homologous proteins (figs. S1 and S7, C and D).

### Dynamic motions in state interconversions

**Fig. 2.**
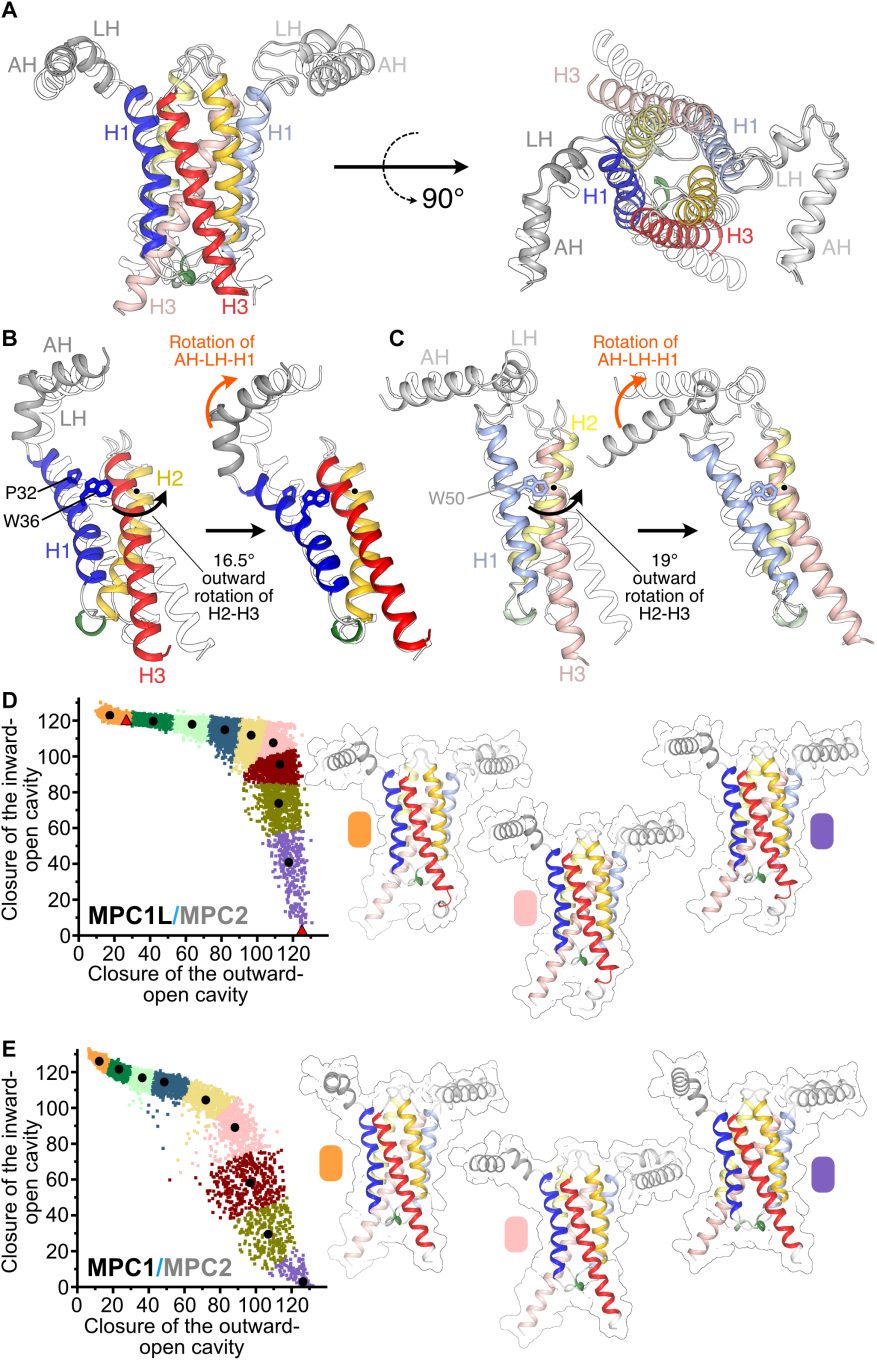
The dynamic motions of the MPC. (**A**) Alignment of human MPC in the outward-open state (colored cartoon) and inward-open state (outline). (**B**) Overview of MPC1L in the outward-open (colored cartoon) and inward-open (outline) states before (left) and after (right) alignment of H2 and H3, viewed down the rotation axis (black dot and curved arrow). Residues flanking the hinge region in H1 are shown in stick representation and are labeled. (**C**) Overview of MPC2 in the outward-open (colored cartoon) and inward-open (outline) states before (left) and after (right) alignment of H2 and H3, as in (B). (**D**) Dynamics of human MPC1L/MPC2 modeled with AlphaFold 2.0. Dots represent individual models clustered according to the degree of inward and outward closure with the occluded states in the middle. Each most centrally located model of the cluster is depicted with a black dot and labeled with the color of their cluster. The models closest to the experimentally determined structures are indicated with a red triangle. The structures of the center of three clusters are also shown. (**E**) Dynamics of human MPC1/MPC2, as in (D).

Rigid-body alignment of the two states reveals distinct conformational changes consistent with an alternating access mechanism of transport (Fig. 2 and movie S1). In the outward-open to inward-open state conversion, H2 and H3 significantly move outward on the matrix side, opening up the central cavity to the matrix, whereas smaller movements on the intermembrane side close access to the central cavity from the intermembrane space.

Aligning each subunit separately shows that the conformational change consists of a rigid-body rotation of the H2 and H3 helical bundle by 16.5° for MPC1L and 19° for MPC2 (Fig. 2, B and C). This rotation is combined with the bending of H1 to avoid clashing with H2 of the other subunit. For MPC1L, the bending region in H1 is localized and runs from P32 to W36, which are both highly conserved. P32 and nearby G31 and G37 likely contribute to the bending of H1 (Fig. 2B). The bending region for MPC2 is more extensive, running from the conserved W50 to the loop between H1 and H2 (Fig. 2C). Conserved G51 and G56 in this region of MPC2 are likely to accommodate the bending of H1. Repositioning of the linker and amphipathic helices is coupled to the bending of H1. While the observed conformational changes are distributed asymmetrically across MPC, key residues, such as H86 (MPC1L), K49 (MPC2), and W82 (MPC2), are located in a region that undergoes relatively minor changes, close to the rotation axes for the H1-H2 helical bundles.

The two determined structures reflect endpoints in the alternating access mechanism that covers a wider conformational landscape. To explore the MPC conformations, we generated a large number of models of the MPC1L/MPC2 and MPC1/MPC2 heterodimers with AlphaFold multimer by varying the depth of multiple sequence alignment because it has been shown previously that the variation of restraints obtained with this approach increase the sampled conformational space (Fig. 2, D and E) (*33*). Notably, this strategy generated MPC1L/MPC2 models similar to both experimentally determined state structures, with RMSD values of 0.5 Å for the outward-open state and 1.3 Å for the inward-open state for the transmembrane Cα atoms. Moreover, we obtained models that cover the entire conformational space between the two endpoint structures with the opening and closing of the two pathways quantified using a metric of residue packing. Based on this metric, we grouped the models in different conformational states and identified representative models closest to the center of each cluster (Fig. 2D). These representatives are, on average, <1-Å RMSD from all other models in that cluster, and, thus, the models within each group are structurally similar. The representative models clearly show that the transporter moves gradually from an outward-open to an inward-open state via an occluded state, which is not open to either side of the membrane (movie S2). Moreover, similar open and occluded conformational states connected by a transitional landscape were also obtained for the MPC1/MPC2 dimer (Fig. 2E).

### The pyruvate binding site of the MPC heterodimer

We attempted to solve the structure of a pyruvate-bound state. At pH 7.0 with 10 mM pyruvate, 60% of particles were in an inward-open conformation (3.7- to 4.0-Å resolution) and 40% in an outward-open conformation (>6-Å resolution), indicating that the PMb is unable to hold MPC in a fixed state, which we confirmed independently by showing that bound nanobody does not prevent transport (fig. S14). We also tried using 200 mM pyruvate at pH 5.5, which resulted in an outward-open conformation (3.5- to 3.7-Å resolution) very similar to the mitoglitazone-bound structure. However, a density consistent with the molecule pyruvate was not observed in any of these cryo-EM maps. These results show that MPC behaves as expected because pyruvate binding must lead to conformational changes and substrate release. Human MPC is a low substrate affinity transporter, as can be deduced from its apparent Michaelis constant (*K*_m_) of transport of 200 ± 53 μM (three technical repeats). A high substrate affinity is not required because the high transport rates of MPC guarantee an efficient substrate translocation and support the notion that conformational changes occur quickly. Thus, MPC moves extremely fast through the pyruvate-binding transition states, meaning that the substrate-bound outward-open and inward-open states will have very low occupancies.

For this reason, we applied several other approaches to locate the pyruvate binding site. The occluded state models of both heterodimers contain a small central pocket, which could bind pyruvate. In the MPC1L/MPC2 occluded model, this pocket is lined by the same residues present at the bottom of the cavities in the outward-open and inward-open state structures (Fig. 1, B to E). These residues are S35, L38, Y64, F68, F71, L82, and H86 (MPC1L) and K49, T78, W82, N100, and L96 (MPC2). To locate the pyruvate binding site, we introduced single alanine mutations to assess pyruvate binding (fig. S15). Of these variants, only N100A could not be produced in a folded state. To assess whether these mutants were functional, we first tested their transport activities in proteoliposomes (fig. S16). The transport activities of all variants were severely affected, showing that most of these residues are essential for pyruvate transport, except for S35A (fig. S16).

**Fig. 3.**
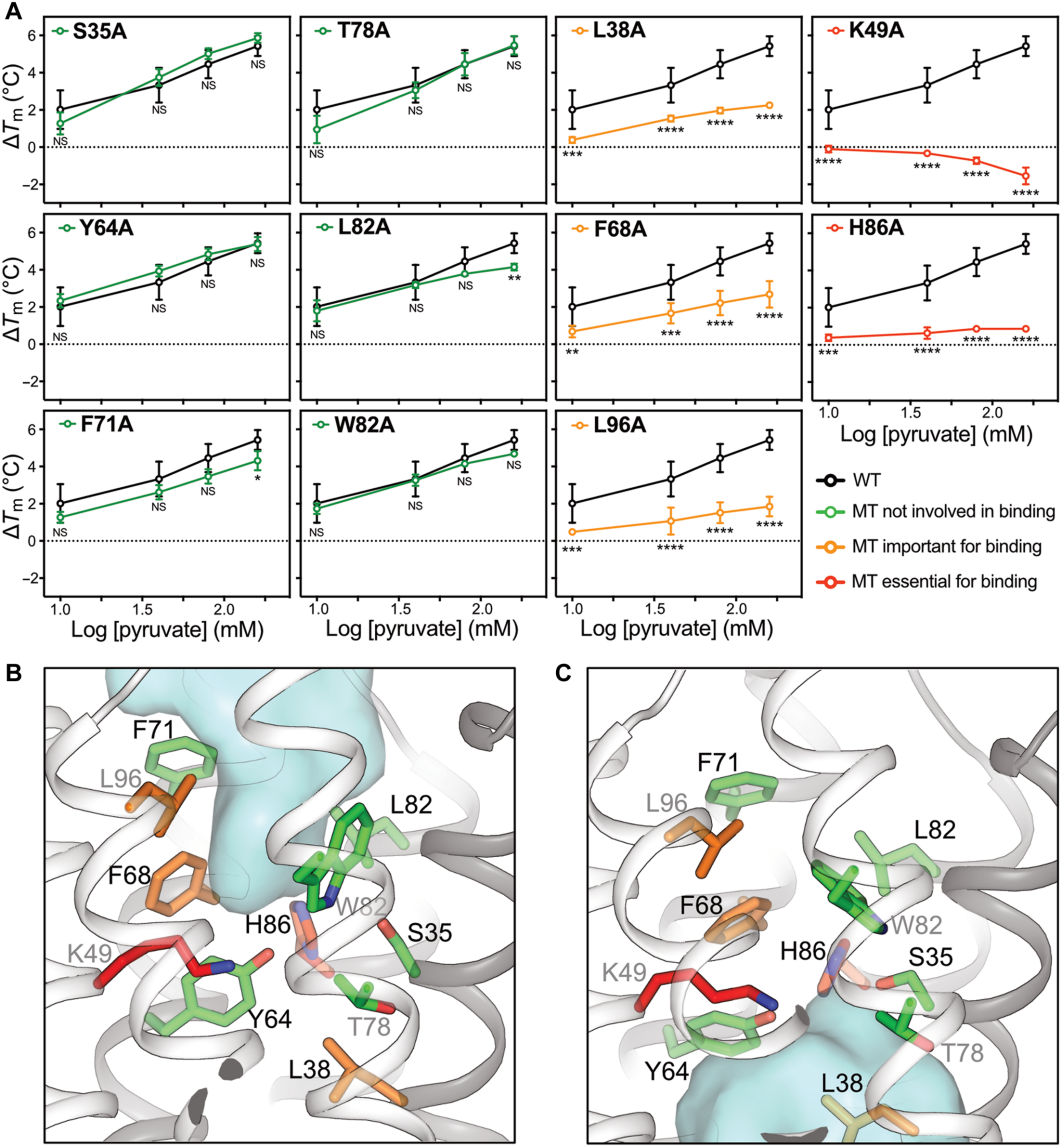
Mapping the location of the pyruvate binding site. (**A**) Screening of cavity residues for pyruvate binding. Thermostability shifts (∆*T*_m_) for wild-type MPC (black line) and 11 single alanine replacement mutants (colored lines, legend) are shown in the presence of 10, 40, 80, and 160 mM pyruvate. Error bars show SD across three biological replicates [not significant (NS), *P* > 0.05; *P < 0.05; **P < 0.01; ***P < 0.001; ****P < 0.0001]. WT, wild type; MT, mutant. Pyruvate-binding site in the (**B**) outward-open state and (**C**) inward-open state with residues colored according to their importance for binding, as in (A). MPC1L and MPC2 are shown as dark and light gray cartoons, respectively. The water-accessible cavities are shown as blue surfaces.

**Fig. 4.**
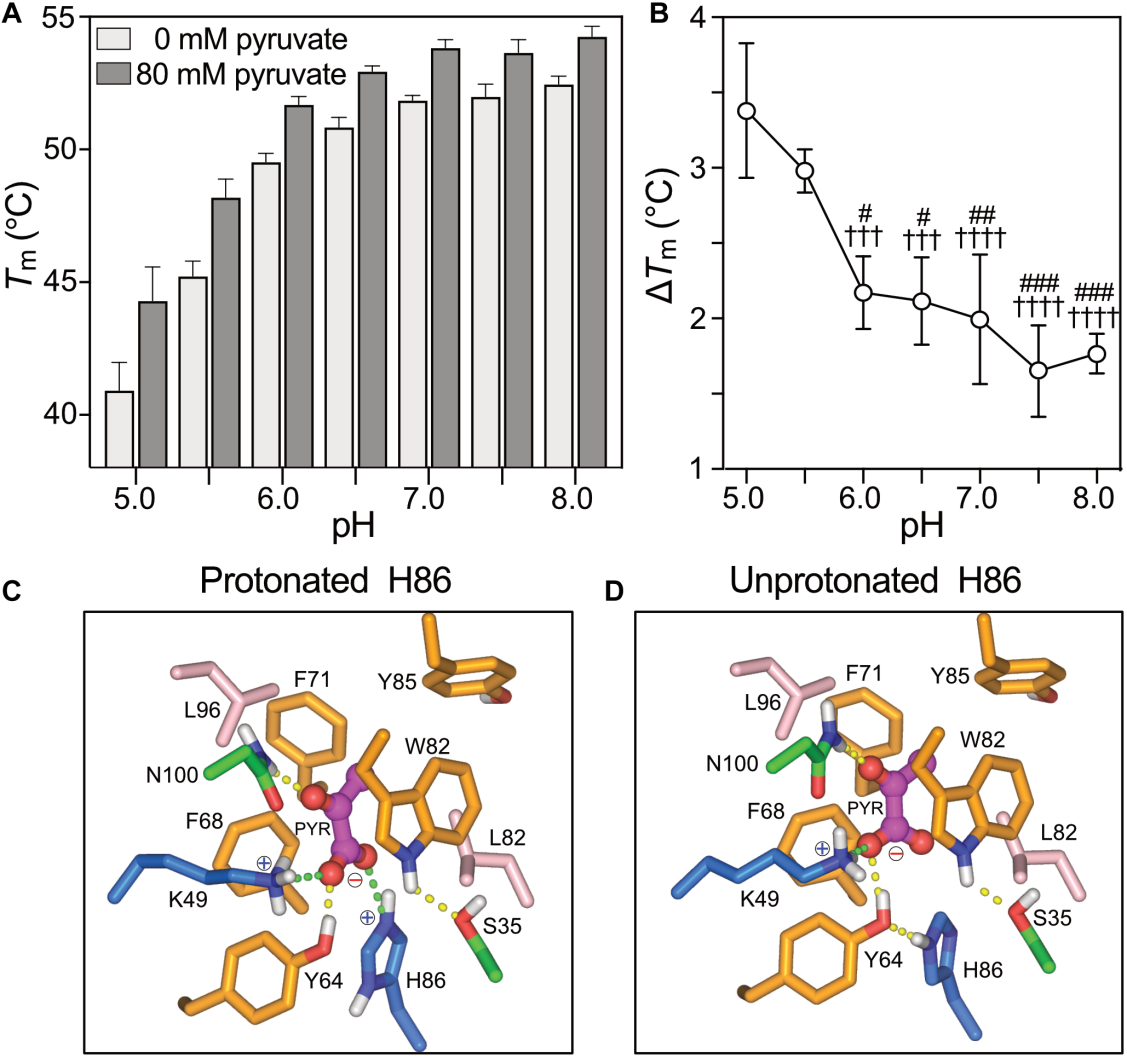
The pH dependency of pyruvate binding. Pyruvate binding to wild-type MPC1L/MPC2 in a range of pH values, measured by nanoDSF. (**A**) Apparent melting temperatures (*T*_m_) and (**B**) thermostability shift (∆*T*_m_) at 80 mM pyruvate. Error bars show SD across four biological replicates. †††*P* < 0.001 and ††††*P* < 0.0001 compared to ∆*T*_m_ at pH 5.0; #*P* < 0.05, ##*P* < 0.01, ###*P* < 0.001 compared to ∆*T*_m_ at pH 5.5. (**C** and **D**) Docking studies of pyruvate (PYR) in the representative occluded state of MPC1L/MPC2 (fig. S17), with H86 either protonated (C) or neutral (D). Residues are colored according to the Zappo scheme with salt bridge and hydrogen bond interactions shown as green and yellow dashed lines, respectively.

Next, we determined which of these residues are specifically important for substrate binding. We have previously established that thermostability shift assays can be used to map inhibitor and substrate binding sites of transport proteins (*34*, *35*). In wild-type MPC, pyruvate binding causes thermostability shifts in a concentration-dependent manner (Fig. 3A, black lines). Single alanine replacements of residues K49 and H86 fully abolished the concentration-dependent thermostability shift (Fig. 3A, red lines), indicating that they form critical interactions with pyruvate. The L96A, L38A, and F68A mutations had a modest but statistically significant effect on pyruvate binding (Fig. 3A, orange lines). These residues are accessible in the water-filled cavity in the outward-open state (Fig. 3B) and in the inward-open state (Fig. 3C). Next, we measured for wild-type MPC thermostability shifts at various pH values in the presence of 80 mM pyruvate (Fig. 4, A and B). The thermostability of the MPC complex increased with increasing pH values. The shifts, which are the consequence of pyruvate binding, are the largest at pH 5 and 5.5 but decrease significantly at higher pH values, showing that pyruvate binding is pH-dependent with a transition around the p*K*_a_ (where *K*a is the acid dissociation constant) of histidine, implicating H86 (Fig. 4B).

To understand the pH dependency of pyruvate binding better, we docked pyruvate into the representative occluded state model of MPC1L/MPC2, where the interactions are expected to be optimal (Fig. 4, C and D; fig. S17; and movie S2) while allowing the residues of the binding site to rearrange. Two binding studies were carried out to account for the observed pH effect (Fig. 3B), in which H86 was modeled as either protonated (Fig. 4C) or neutral (Fig. 4D). A significant number of predicted pyruvate poses were obtained, independent of the protonation state of H86 (70 and 67 of a total of 80 possible poses, respectively), indicating that pyruvate can bind in the small pocket in the occluded state model.

Each docking study yielded a converged set of protein-ligand interactions (80 and 70% of all predicted poses). In the protonated form, H86, together with K49, binds to the conjugated and negatively charged carboxylic group of pyruvate via ionic interactions (Fig. 4C). The hydroxyl group of Y64 is predicted to donate an additional hydrogen bond to the carboxylic group. N100 donates a hydrogen bond to the ketone group of pyruvate. The carbon backbone of pyruvate is sandwiched between the aromatic F68 and W82, forming van der Waals interactions. Last, the methyl group of pyruvate is bound in a hydrophobic pocket formed by Y85, F71, and L96. This bonding arrangement is supported by the pyruvate thermostability assay (Fig. 3A), which shows that H86 and K49 are essential and L96 and F68 are important for binding. The only unexpected result is that Y64 is not important for binding (Fig. 3A). Still, it is possible that the tight binding to the essential H86 and K49 is sufficient or that the Y64 side-chain region can be occupied by water without significant energetic cost to binding. All other residues are binding weakly and thus might not show an effect. When H86 is neutral, it forms a hydrogen bond with Y64 rather than with pyruvate, decreasing the overall number of interaction bonds (Fig. 4D).

### The structural basis of binding of UK5099 and its derivatives

**Fig. 5.**
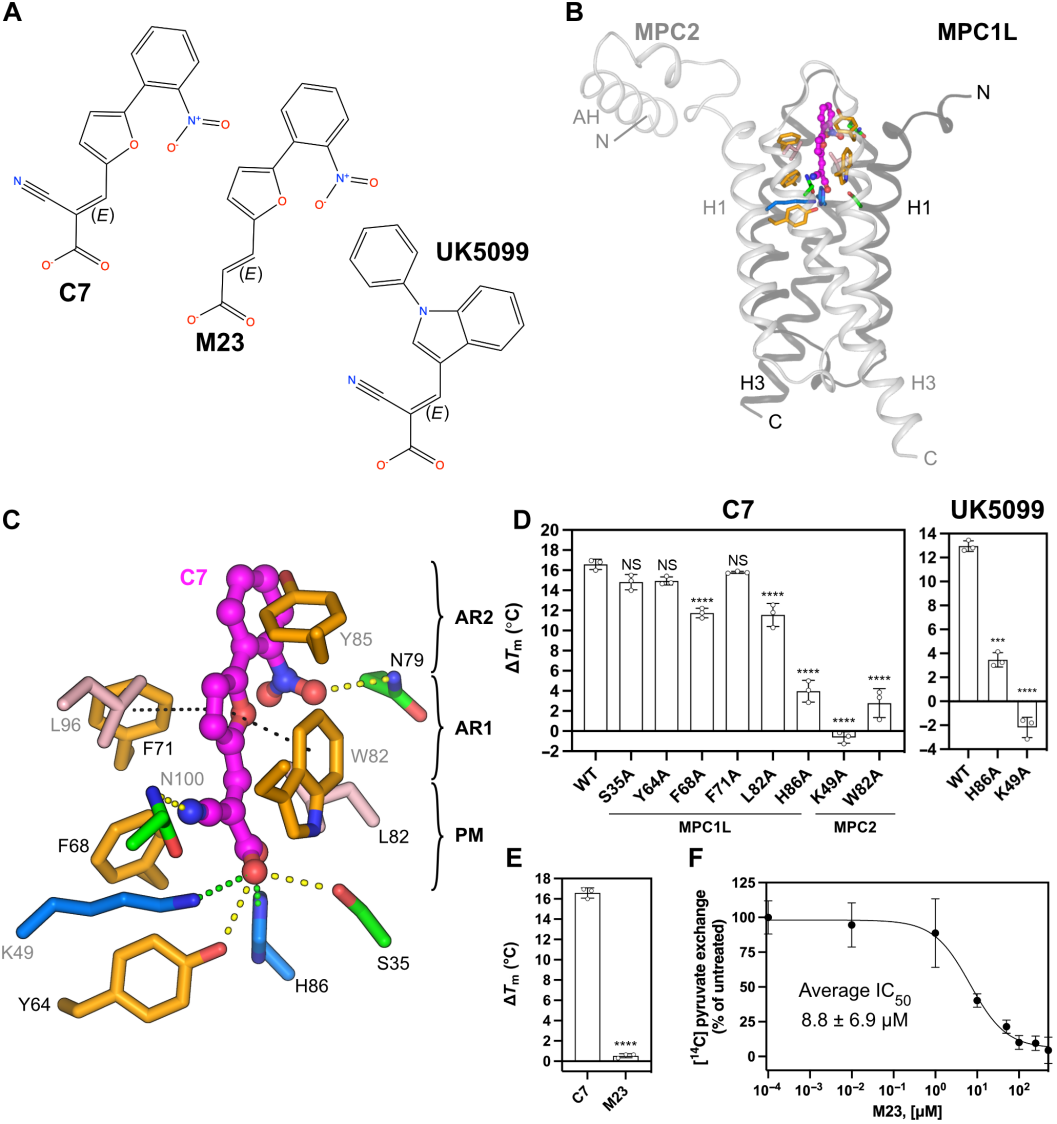
The binding site of a UK5099 analog. (**A**) Chemical structures of UK5099, its derivative C7 and compound M23, with stereochemistry indicated. (**B**) Overview of the inhibitor-binding site. MPC is shown as a cartoon, with MPC1L in dark gray and MPC2 in light gray. Helices and N and C termini of MPC1L and MPC2 are labeled. C7 is shown with magenta carbon atoms. Amino acid residues forming the binding pocket are shown in stick representation with the Zappo color scheme. (**C**) Detailed view of the binding site with salt bridge, hydrogen bond, and hydrophobic π-stacking interactions as green, yellow, and black dashed lines, respectively. Key features of the inhibitor are indicated: PM, pyruvate-mimic; AR1, aromatic ring 1; AR2, aromatic ring 2. (**D** and **E**) Thermostability shift assays in the presence of 100 μM inhibitor. The temperature shift (Δ*T*_m_) is the apparent melting temperature in the presence of a compound minus the apparent melting temperature in the absence of compound. The data represent the mean and SD of three independent experiments: ***P < 0.001; ****P < 0.0001; NS, *P* > 0.05. (**F**) Inhibition of [^14^C]-pyruvate exchange in MPC1L/MPC2 proteoliposomes by compound M23. The data represent the mean and SD of three independent experiments.

Alpha-cyano-cinnamates and their derivatives, which include UK5099 (IC_50_ of 50 nM), were the first described MPC inhibitors (*8*, *25*). We have recently developed non-indole derivatives that maintain the cyano and carboxyl moieties (cyanoacrylate). The tightest binder was (*E*)-2-cyano-3-[5-(2-nitrophenyl)-2-furyl]acrylic acid, called C7 (Fig. 5A), which has an IC_50_ of 5 nM (*14*), and was used to solve a cryo-EM structure of inhibited MPC (figs. S18 and S19 and table S1). The resolution as determined by gold-standard FSC was 3.65 Å but, for the transport domain, was in the region of 3.1 to 3.5 Å (fig. S5C), and the amphipathic helices and C termini had lower resolutions, as they are naturally flexible (fig. S10). A C7 molecule could be modeled in an extra density (fig. S20A) and binds in a narrow crevice between MPC1L and MPC2 protomers, oriented with the cyanoacrylate group, which mimics pyruvate, facing the bottom of the cavity (Fig. 5B). The carboxylate group forms an ionic interaction with H86 (MPC1L) and K49 (MPC2) and hydrogen bonds with S35 and Y64 (MPC1L). We investigated the impact of alanine replacements on C7 binding using thermostability shift assays. K49A eliminated the 16.5° ± 0.4°C shift observed with wild-type MPC, confirming that it is essential (Fig. 5D). H86A also reduced the shift markedly to 4.0° ± 1.1°C. UK5099 binding was also abolished in K49A and H86A mutants (Fig. 5D). Disruption of the hydrogen bonds with S35 and Y64 was well tolerated, with S35A and Y64A showing the same shift as wild-type MPC within error. F68A reduced the thermostability shift to 11.7° ± 0.4°C. The cyano group forms a hydrogen bond with N100 (MPC2) (Fig. 5C and fig. S21A), but N100A could not be purified. However, when we assayed a derivative of C7, (*E*)-3-[5-(2-nitrophenyl)-2-furyl]-acrylic acid (M23), which lacks this group (Fig. 5A), it did not generate a shift with wild-type MPC (Fig. 5E). Its inhibitory potency was more than a thousand times lower than that of C7 (Fig. 5F), demonstrating the importance of the cyano group in binding of C7.

The furan ring of C7, which is an indole in UK5099, forms perpendicular π-stacking interactions with F71 (MPC1L) and parallel π-stacking interactions with W82 (MPC2) (Fig. 5C and fig. S21A). W82A abolished the thermostability shift by C7, whereas F71A did not have a significant effect (Fig. 5D). This observation shows that W82 plays an essential role, not only by coordinating the furan ring but also in the positioning of the cyanoacrylate group. No aromatic stacking interactions were observed with the second aromatic ring, but the nitro group at position R1 forms a hydrogen bond with N79 (MPC1L). Consistent with this additional interaction forming, when this nitro group is removed, the inhibitory potency is reduced twofold, and, when it is moved to the R2 position, a decrease in the thermostability shift had been observed, confirming the importance of this interaction (*14*).

The interaction of alpha-cyano-cinnamates and their derivatives with MPC has been proposed to be mediated by a covalent bond formed after a Michael reaction between their activated double bond and a cysteine residue of MPC. However, the structure shows that the closest cysteine, C85 (MPC1L), is 9.4 Å away from the double bond, which is too far to react.

### The structural basis of TZD binding

TZDs are PPARγ agonists that have been used for treating type 2 diabetes, but they also inhibit MPC with low micromolar potencies (2.7 μM) (*14*). New-generation TZDs with decreased affinity for PPARγ maintain the therapeutic benefit (*36*, *37*) and have been considered in clinical trials for MASH (*17*, *38*) and Alzheimer’s disease (*39*). A deuterium-modified (*R*)-pioglitazone lacking in vitro activity for PPARγ has entered clinical trials for treating MASH (*18*).

**Fig. 6.**
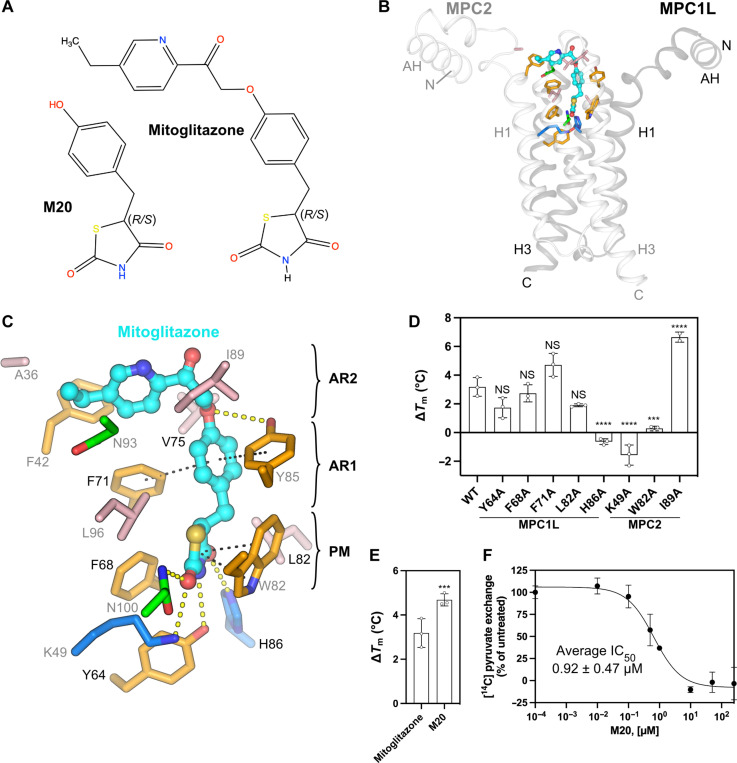
The binding site of the TZD mitoglitazone. (**A**) Structure of mitoglitazone and the shorter TZD (*E*)-5-(4-hydroxybenzylidene) thiazolidine-2,4-dione (M20), with stereochemistry indicated. (**B**) Overview of the inhibitor-binding site, as in Fig. 4B. (*S*)-mitoglitazone is shown with cyan carbon atoms. (**C**) Detailed view of the binding site, with hydrogen bonds shown as yellow dashed lines and hydrophobic π-stacking interactions as black dashed lines, as in Fig. 4C. (**D** and **E**) Thermostability shift assays in the presence or absence of 100 μM inhibitor, shown as in Fig. 4 (D and E). ***P < 0.001, ****P < 0.0001 (**F**) Inhibition of [^14^C]-pyruvate exchange in MPC1L/MPC2 proteoliposomes by compound M20, shown as in Fig. 4F. The data represent the mean and SD of three independent experiments.

We solved the structure of MPC with mitoglitazone (MSDC-0160, 5-[[4-[2-(5-ethyl-2-pyridinyl)-2-oxoethoxy]phenyl]methyl]-2,4-TZD) (Fig. 1, A to C; Fig. 6A; figs. S4 and S6; and table S1). Mitoglitazone could be modeled into a density (fig. S20B), found at the same place as C7, but with different interactions (Fig. 6, B and C). The C5 position of the TZD ring contains a chiral center (Fig. 6A) with a known propensity to racemize via keto-enol tautomerism (*40*). Consistent with this, the cryo-EM density map is compatible with either stereoisomer (fig. S6C), and we have modeled both with 50% occupancy. Most interactions involve the TZD ring, which acts as a pyruvate-like moiety (Fig. 6C and fig. S21, B and C). The nitrogen of the TZD ring forms a hydrogen bond with Y64, whereas the oxygen atoms form hydrogen bonds with H86 (MPC1L) and K49 and N100 (MPC2). The number and strength of hydrogen bond interactions between the TZD ring and MPC are similar for both stereoisomers (fig. S21, B and C). In agreement, K49A and H86A mutations eliminated the thermostability shift seen in wild-type MPC with mitoglitazone (Fig. 6D). The TZD ring also forms parallel 𝜋-stacking interactions with W82 (MPC2) (Fig. 5C and fig. S21, B and C) and W82A eliminated the shift (Fig. 6D). The proximal residues F68 and L82 had no significant effect on mitoglitazone binding, as their mutation did not generate a change in shift.

Beyond the TZD ring, the phenyl ring forms perpendicular 𝜋-stacking interactions with F71 (MPC1L) and Y85 (MPC2), but F71A did not affect the thermostability shift (Fig. 6C and fig. S21, B and C). The electron density is weaker for the ether linker but is consistent with the placement of the pyridine ring, which sits on the solvent-exposed intermembrane space surface of MPC (Fig. 6, B and C, and fig. S20B). The terminal ethyl group is positioned between the H1 and H2-H3 loop of MPC2, where A36, F42, I89, and N93 are involved in hydrophobic contacts (Fig. 5C and fig. S21, B and C). A weak hydrogen bond may form between the ether oxygen and hydroxyl of Y85 (MPC2). These weaker interactions are consistent with higher dynamics and, therefore, weaker cryo-EM map features (fig. S20B).

I89A significantly increased the thermostability shift induced by mitoglitazone binding compared to wild-type MPC (Fig. 6D), suggesting stronger binding. This observation, combined with the placement of this part of the molecule in the intermembrane space, raises the question of whether the pocket is too small to accommodate TZDs optimally. Therefore, we tested the binding and inhibitory potency of a shorter TZD, (*E*)-5-(4-hydroxybenzylidene)thiazolidine-2,4-dione (M20) (Fig. 6A). The thermostability shift elicited by M20 was 1.5° ± 0.5°C higher than by mitoglitazone (*P* < 0.001) (Fig. 6E), and its inhibitory potency was approximately three times higher (Fig. 6F). These results emphasize that the thiazolidine-2,4-dione and benzene groups mediate all of the essential interactions (*1*, *14*) and that the terminal aromatic ring is not required for high-affinity binding.

### The structural basis of zaprinast binding

**Fig. 7.**
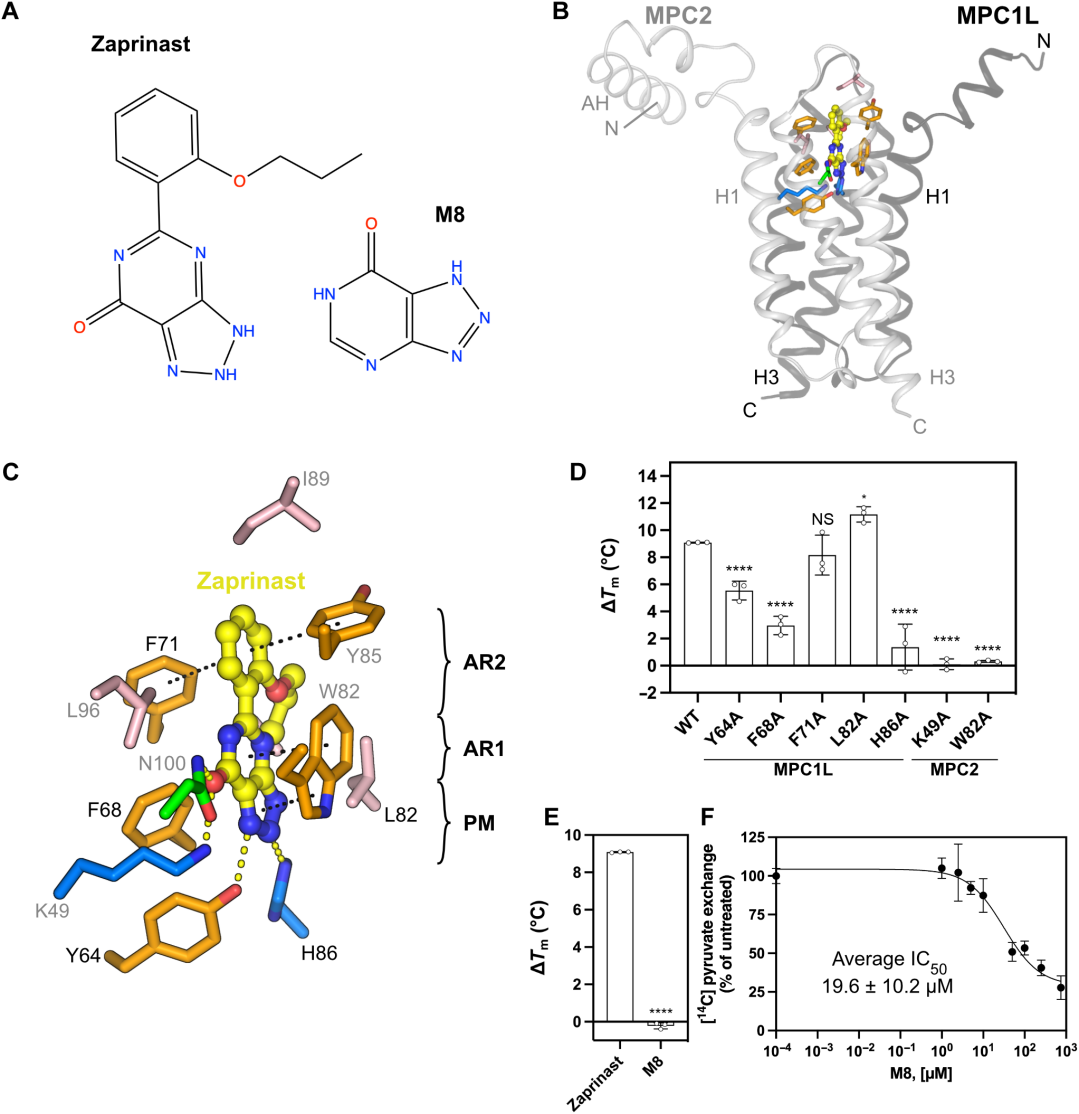
The binding site of zaprinast. (**A**) Chemical structure of zaprinast and the derivative M8. (**B**) Overview of the inhibitor-binding site, shown as in Fig. 4B. Zaprinast is shown with yellow carbon atoms. (**C**) Detailed view of the binding site, with hydrogen bonds shown as yellow dashed lines, and hydrophobic π-stacking interactions as black dashed lines, as in Fig. 4C. (**D** and **E**) Thermostability shift assays in the presence or absence of 100 μM inhibitor, as in Fig. 4 (D and E). *P < 0.05, ****P < 0.0001 (**F**) Inhibition of [^14^C]-pyruvate exchange in MPC1L/MPC2 proteoliposomes by compound M8, shown as in Fig. 4F. The data represent the mean and SD of three independent experiments.

Last, we studied zaprinast [5-(2-propoxyphenyl)-2,6-dihydrotriazolo[4,5-d]pyrimidin-7-one] binding (Fig. 7A), a phosphodiesterase inhibitor and lead compound in the development of sildenafil (Viagra), which inhibits pyruvate-driven oxygen consumption in brain mitochondria and pyruvate uptake in isolated mitochondria (*19*, *41*). Zaprinast inhibits pyruvate transport in reconstituted human MPC1L/MPC2 with high potency (321 nM) (*14*).

The cryo-EM structure with bound zaprinast (Fig. 7B; figs. S20C, S22, and S23; and table S1) shows that the inhibitor binds to the same site as C7 and mitoglitazone in the outward-open state (Fig. 6, B and C). The resolution as determined by gold-standard FSC was 3.92 Å but the transport domain was 3.3 to 3.5 Å, and lower for the amphipathic helices and C termini (fig. S5D), which are naturally flexible. Its triazolopyrimidinone ring acts as a pyruvate-mimic and is close to where the cyanoacrylate and TZD groups bind. The heterocyclic ring provides hydrogen bond donors and acceptors to Y64 and H86 (MPC1L) and K49 and N100 (MPC2) (Fig. 7C and fig. S21D). H86A markedly reduced the zaprinast-induced thermostability shift, from 9.1° ± 0.2°C to 1.4° ± 1.4°C, whereas K49A eliminated it altogether (Fig. 7D). F68A forms van der Waals interactions with the heterocyclic ring, and its alanine replacement mutant had a significantly reduced shift. W82 forms parallel π-stacking interactions with the triazolopyrimidinone ring (Fig. 7C and fig. S21D), and W82A showed no shift with zaprinast, as with other inhibitors (Figs. 5 and 6). F71 (MPC1L) and Y85 (MPC2) form perpendicular π-stacking interactions with the phenyl group (Fig. 7C and fig. S21D), but F71A exhibited a similar shift as wild-type MPC upon zaprinast binding. However, the phenyl ring is essential for high-affinity binding, as shown using the derivative 8-azahypoxanthine (Fig. 7A), which gave no shift (Fig. 7E) and had an inhibitory potency (19.6 ± 10.2 μM), ~60 times lower than zaprinast (Fig. 7F).

## DISCUSSION

Here, we provide structures of the human MPC in the outward-open and inward-open configurations, unliganded or liganded with three different classes of inhibitors. Although the FSC values were 3.6 to 3.9 Å, being negatively affected by the flexible positions of the amphipathic helices, the transport domain of MPC has a resolution of 3.2 to 3.5 Å, which was sufficiently informative to build atomic models. These structures resolve several fundamental aspects of MPC structure and mechanism. First, they demonstrate that functional MPC, capable of binding substrate and inhibitors, is a heterodimer, as previously proposed (*13*, *14*). Second, they provide structural evidence for the helical topology, showing that both protomers consist of an N-terminal amphipathic helix, a linker helix, and three transmembrane helices, in contrast to the different topologies proposed for each protomer of yeast and human MPC (*10*, *12*). We have also determined the orientation of MPC in native membranes and found that the N-terminal amphipathic helices are located in the intermembrane space and the C-terminal region in the matrix, in contrast to other proposals (*10*, *12*). In our proposed orientation, the inhibitors could pass through the mitochondrial outer membrane via voltage-gated anion channels before they inactivate MPC by binding to the outward-open state. If the orientation were to be inverted, as suggested previously (*10*, *12*), then the inhibitors would have to pass through the protein-dense mitochondrial inner membrane by passive diffusion first before they could act on MPC. Our proposed orientation is compatible with a TIM22-mediated insertion mechanism, which leaves the N terminus in the intermembrane space (*11*).

MPC is one of the smallest transporters in nature, forming a functional heterodimer with only six transmembrane helices. Despite their name, MPC proteins (SLC54) are completely unrelated in structure and mechanism to members of the SLC25 mitochondrial carrier family, which are threefold pseudosymmetrical and function as monomers (*42*–*45*). MPC belongs to the Transporter-Opsin-G protein–coupled receptor superfamily (*46*), which also contains the semiSWEET (PDB code: 4QND) (*47*), SWEET (PDB code: 5XPD) (*48*), and cystinosin (PDB code: 7ZKW) (*49*) transporters. However, the latter two have an extra transmembrane helix linking the domains (fig. S24). MPC has the closest structural resemblance to the bacterial semiSWEET homodimer (RMSD of 6.2 Å), which transports sugars (*47*, *50*, *51*), although there is no sequence similarity. Typical for members of this superfamily, the transmembrane helical arrangement of MPC is not sequential, but, instead, H3 is placed between H1 and H2. An unusual feature of MPC is the presence of long N-terminal amphipathic helices, linked by a linker helix to the transmembrane complex (Fig. 1). The MPC1L/MPC2 heterodimer is twofold pseudosymmetrical from the N-terminal linker helix to the C-terminal H3. The symmetry is broken by the N-terminal amphipathic helices, which are arranged in parallel but with the N termini pointing the same way. The inversion of the amphipathic helix of MPC2 is due to a conserved interaction network between residues D18, E21, N33, and R39, which form a sharp helix-turn-helix motif (fig. S8A). The amphipathic nature of the linker and the amphipathic helices can be deduced from the distribution of polar and hydrophobic residues and electrostatic surface potential (fig. S8, A and B). The hydrophobic side will interact with the hydrophobic core of the inner membrane. In contrast, the polar side contains many arginine and lysine residues, which can snorkel to the negative charges in the headgroup layer (*52*). The amphipathic helices are oriented approximately perpendicular to the dimerization interface (Fig. 1, A and D, and figs. S8 and S10), allowing the transport domain to change conformation unopposed.

By using the outward-open and inward-open structures, as well as computational analyses and functional data, we propose a conformational mechanism and provide a mechanistic rationale for ΔpH-driven pyruvate transport for MPC. The structures show that MPC operates via an alternating access rocker-switch mechanism (*53*), which also has some resemblance to that of the bacterial semiSWEET transporters (*47*, *50*, *51*), although they share no sequence homology. In the latter, H1 kinks at a highly conserved PQ-loop motif, dividing it into two helical parts. Conformational changes between states involve part of H1, along with H2 and H3, moving as a rigid body, similar to the motions observed for MPC. We have also defined the conformational space of the MPC1L/MPC2 and MPC1/MPC2 heterodimers, showing that they move progressively from the outward-open to the inward-open state via an occluded state in an alternating access mechanism (Fig. 2, E and F).

MPC has a central substrate binding site with two essential residues, H86 (MPC1L) and K49 (MPC2), accessible at the bottom of the cavities in both states. MPC also has two hydrophobic gates on either side, ~9 Å thick, which regulate access to that site in an alternating way. Pyruvate has a p*K*_a_ of ~2.5, retaining a negative charge under physiological conditions, and, thus, the membrane potential will oppose its passive diffusion into mitochondria. Pyruvate import by MPC depends on the ΔpH, but not on the membrane potential (*13*–*15*), and, thus, should be electroneutral. The essential K49 (MPC2) is always positively charged, whereas H86 (MPC1L) can be neutral or positively charged. We show that pyruvate binding is pH dependent with an inflection point just below pH 6.0 (Fig. 4B), corresponding to the p*K*_a_ for histidine. Docking studies show that the pyruvate bonding arrangement changes depending on the protonation state of H86 (MPC1L) (Fig. 4, C and D). Thus, H86 is likely to be a key component of the ΔpH-driven transport process.

**Fig. 8.**
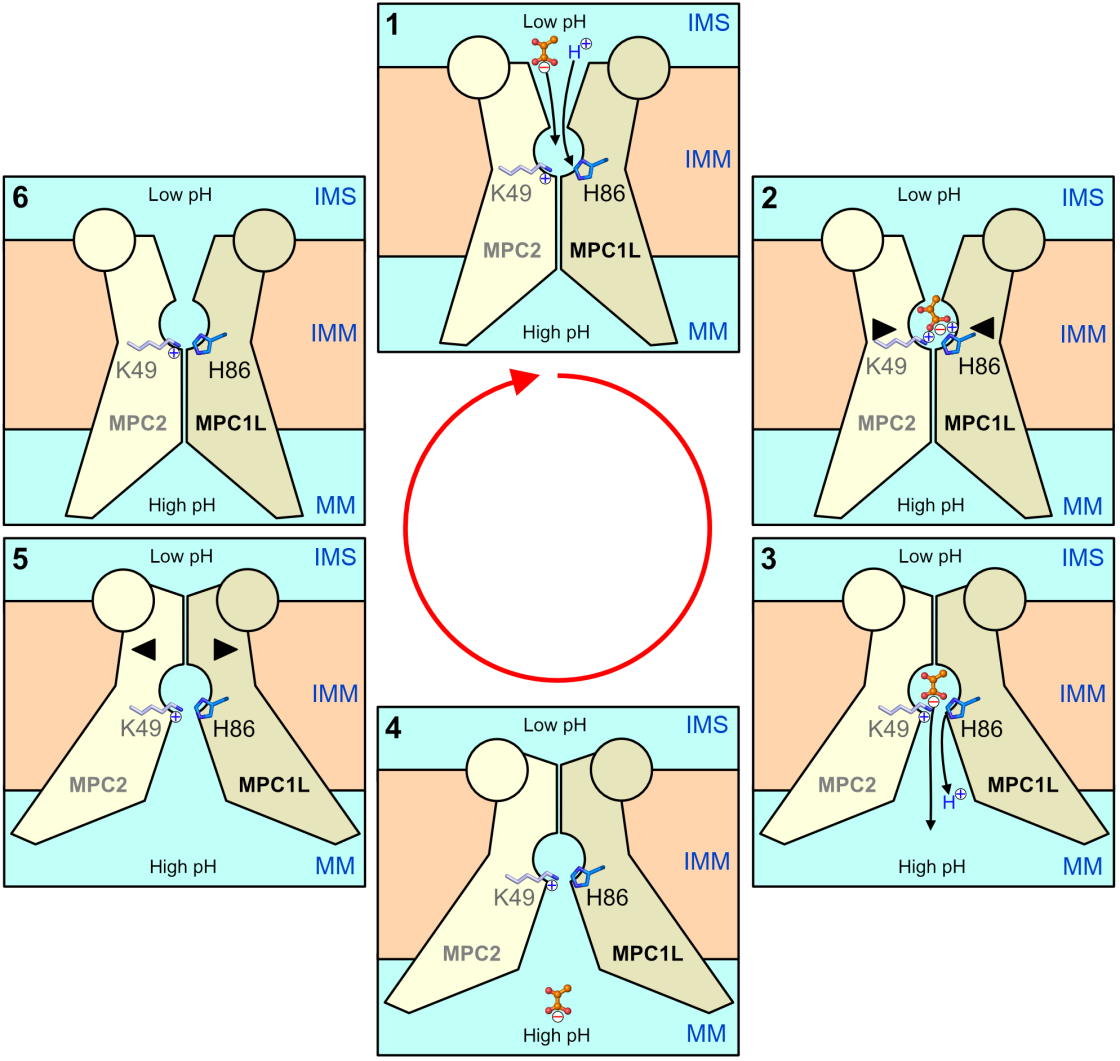
ΔpH-dependent transport mechanism of the MPC. Schematic representation of the pH-dependent pyruvate import mechanism in six stages. The two subunits of MPC are shown schematically with MPC1L in a darker shade and MPC2 in a lighter shade. The circles represent the N-terminal amphipathic helices, which are in the intermembrane space. IMS, intermembrane space; IMM, inner mitochondrial membrane; MM, mitochondrial matrix. The essential residues for pyruvate binding, K49 and H86 (blue), and the substrate (orange) are schematically represented to show their proposed roles.

Conformational changes of transporters occur under the influence of thermal energy (*54*). We propose the following transport mechanism for MPC, which explains its ΔpH-dependency (Fig. 8) (*13*, *15*). In the outward-open state, the binding site is exposed to the lower pH of the intermembrane space, and the positively charged K49 will attract pyruvate while simultaneously shifting the p*K*_a_ of H86 to bind a proton (Figs. 8 and 3E). The interaction with pyruvate might move K49 on MPC2 and H86 on MPC1L closer together, initiating the conformational changes to the inward-open state. The binding site is now exposed to the higher pH environment of the matrix, which would enable deprotonation of H86 and rearrangement of the pyruvate bonding (Fig. 4D), which would allow it to leave the binding site. Under the influence of thermal energy, the unliganded inward-open state could then rearrange to the outward-open state, as the latter is a lower energy state due to the larger number of inter-subunit interactions (fig. S9). In this way, pyruvate transport by MPC depends on the ΔpH but not on the membrane potential, as no net charge is transported (Fig. 8). The binding pocket of the cystinosin transporter, another member of the Transporter-Opsin-G protein–coupled receptor superfamily (*46*), also uses a histidine and lysine residue, recognizing the two carboxylate ends of cystine (*49*). Furthermore, a His replacement mutant no longer displays a pH-dependent transport activity (*49*), as with MPC.

**Fig. 9.**
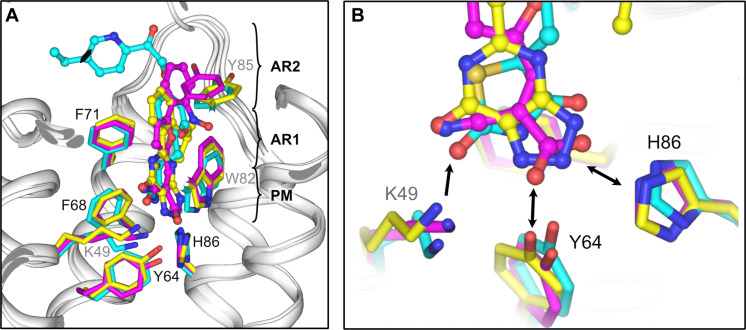
Common principles of inhibitor binding to the MPC. (**A**) Overlay of human MPC inhibited by C7 (magenta), mitoglitazone (cyan), and zaprinast (yellow). PM, pyruvate mimic; AR1, first aromatic ring; AR2, second aromatic ring. (**B**) Close-up view of the PM region. Y64 and H86 (MPC1L) and K49 (MPC2) act as hydrogen bond donors (single arrowhead) or donors/acceptors (double arrowhead). K49 and H86 can also interact via salt bridges, depending upon the inhibitor chemistry.

The mechanism of transport inhibition is of great interest, as MPC is being investigated as a drug target for a range of conditions. Although the chemistries of the three inhibitor classes are completely different, the binding interactions follow similar principles. By adopting planar structures, all inhibitors bind to a narrow cavity, open to the intermembrane space, which lies on the twofold pseudosymmetrical axis of the heterodimer (Fig. 9A). The major interactions involve K49 (MPC2) and H86 (MPC1L), which interact with the pyruvate-mimicking moieties of each inhibitor, as well as W82 (MPC2), which provides aromatic stacking arrangements. Their mutation to alanine eliminates binding across the three tested inhibitor classes. Overall, the extensive network of interactions involves residues from both protomers, supporting previous experimental observations that ligand binding requires the formation of a functional heterodimer (*13*, *14*). These structural observations also explain why a functional binding pocket cannot be formed by homodimers, as suggested previously (*55*). These structures also refute the widely accepted belief that UK5099 and its derivatives bind covalently, forming Michael addition adducts with a cysteine residue (*25*). This notion is consistent with mass spectrometry measurements that did not detect covalent bond formation and single alanine replacements of each cysteine residue, which did not alter the thermostability shift by UK5099 or C7 (*14*). Instead, the high binding affinity of these inhibitors is now fully explained by the extensive network of ionic, polar, hydrophobic, and aromatic interactions.

The observed inhibitor-MPC interactions highlight that the cyanoacrylate group of C7 provides three closely spaced hydrogen bond acceptors, acting as a pyruvate mimic (Fig. 9B) (*14*). The same principle is observed for the TZD ring of mitoglitazone, which provides two hydrogen bond acceptors (carbonyl oxygens) and one donor (amine group), and for the triazolopyrimidinone ring of zaprinast, which provides two hydrogen bond acceptors (carbonyl oxygen and one nitrogen) and one hydrogen bond acceptor or donor (second nitrogen). For all three inhibitors, these acceptor/donor groups lie within a distance of 4.2 to 4.6 Å, mirroring the 3.3-Å distance between the equivalent groups in pyruvate. The interacting residues with pyruvate, K49 and H86, also interact with the key functional groups of the inhibitors (Fig. 9B). Furthermore, a central aromatic group forms π-stacking interactions with MPC, consistent with its importance for high-affinity binding (*14*).

The data presented here have highlighted some key aspects of the ΔpH-dependent pyruvate transport mechanism of MPC. In addition, the binding poses of three major inhibitor classes were revealed, allowing further exploration of MPC as a drug target for treating diabetes mellitus, specific cancers, metabolic dysfunction–associated steatotic liver disease, and neurodegeneration.

## MATERIALS AND METHODS

### Limited proteolysis assay

In total, 3 mg of bovine heart mitochondria, isolated as previously described (*56*), were used to generate mitoplasts by osmotic swelling. First, mitochondria were pelleted by centrifugation and resuspended in 75 μl of 250 mM sucrose, 1 mM EDTA, and 10 mM Hepes-KOH (pH 7.4) before diluting with 20 mM Hepes-KOH (pH 7.4) to a final volume of 1.5 ml and incubated on ice for 15 min. Sucrose and KCl were then added to final concentrations of 250 and 150 mM to prevent further swelling. In parallel, inside-out SMPs from bovine mitochondria were prepared as previously described (*31*).

To establish a limited proteolysis protocol, mitoplasts or SMPs were divided into two halves for incubation without or with proteinase K at a range of concentrations (2.5, 5, 12, 25, and 50 μg/ml) and different incubation times at room temperature. The optimal conditions were 1 min with proteinase K of 2.5 μg/ml for the MPC1 samples, and, for 60 min with proteinase K of 25 μg/ml for the MPC2 and OSCP (oligomycin sensitivity conferring protein) samples, the total volume was 400 μl. The proteinase K digestion was quenched by the addition of 2% phenylmethylsulfonyl fluoride, 2% SDS, and 0.2% laurylmaltose neopentylglycol (LMNG). The samples were analyzed by 16% tris-glycine gels and Western blotting. After transfer, the membranes were first stained by Ponceau S for normalization to the total amount of protein transferred. Western blotting was performed using peptide antibodies against the C termini of MPC1 (anti–MPC1-CTD) or MPC2 (anti–MPC2-CTD) (gift from J. Caroll and J. Walker). A peptide antibody against the OSCP subunit of ATP synthase was used as a control.

### Antibody binding to MPC in bovine SMPs

Bovine SMPs (∼16.6 mg/ml) were thawed and diluted in SMP buffer [10 mM Mops-KOH (pH 7.5) and 250 mM sucrose] to a final reaction volume of 2 ml (1.5 mg of total protein). From this master mix, 200-μl aliquots were removed to serve as negative (no staining) and secondary-only controls. For primary antibody incubation, two 800-μl aliquots were prepared and incubated with 1 μl (1:800 dilution) of either anti–MPC1-CTD or anti–MPC2-CTD antibody (chicken, in-house) at 4°C for 45 min. Following incubation, samples were centrifuged at 40,000 rpm for 30 min to pellet the SMPs, and the pellets were gently resuspended in 800 μl of SMP buffer supplemented with 1% fetal bovine serum. The SMPs were then incubated with 2.5 μl (final concentration of 5 μg/ml) of Alexa Fluor 488–conjugated goat anti-chicken immunoglobulin Y (H+L) secondary antibody (Invitrogen) at 4°C for 45 min, centrifuged again under the same conditions, and resuspended to the sample volume. The labeled SMPs were then analyzed on an Attune NxT flow cytometer (Thermo Fisher Scientific), and the data were processed and visualized using FlowJo.

### Expression and purification of human MPC

The cDNAs for wild-type human MPC protomers MPC1L (UniProt accession code: P0DKB6) and C-terminally His-tagged MPC2 (UniProt: O95563) were cloned in the bidirectional expression vector pBEVY-GU for expression in *S. cerevisiae* as previously described (*13*) with the modification of using strain W303-1B (*MAT*α *leu2-3*,*112 trp1-1 can1-100 ura3-1 ade2-1 his3-11*,*15*). Positive transformants were selected on Sc-Ura/2% glucose plates and grown in 10 liters of the same preculture medium at 30°C for 24 hours before inoculation into 50 liters of YPG/0.1% glucose. Cells were grown at 30°C for 20 hours, induced with 0.4% galactose for 3.5 hours, and then harvested by centrifugation (4000*g*, 20 min, 4°C). Mitochondria were prepared by disrupting cells with a bead mill (Dyno-Mill Multilab, Willy A. Bachofen AG Maschinenfabrik, Switzerland), as previously described (*13*).

The MPC1L/MPC2 complex was purified from isolated mitochondria as previously described (*13*) with buffer modifications. Briefly, 0.5 g of mitochondria were solubilized with 1% LMNG (Anatrace) in 150 mM NaCl, 20 mM bis-tris (pH 7.0), 10% glycerol, and one cOmplete Mini EDTA-free protease inhibitor tablet (Roche) for 1.5 hours at 4°C. After ultracentrifugation (205,000*g*, 45 min, 4°C), the supernatant was collected, followed by batch binding with 0.25 ml of Nickel Sepharose High Performance resin (GE Healthcare) for 2 hours in the presence of 10 mM imidazole. The resin was washed with 10 ml of buffer A [20 mM bis-tris (pH 7.0), 150 mM NaCl, 50 mM imidazole, tetraoleoyl cardiolipin (TOCL; 0.1 mg/ml) (Avanti Polar Lipids), and 0.1% LMNG], followed by 8 ml of buffer B [20 mM bis-tris (pH 7.0), 150 mM NaCl, TOCL (0.1 mg/ml), and 0.1% LMNG], under gravity flow. The Nickel Sepharose was recovered as a slurry; supplemented with 1 mM TCEP [tris(2-carboxyethyl)phosphine] hydrochloride, 50 mM imidazole, and 125 μg of MBP-Tobacco Etch Virus (TEV) protease; and incubated overnight at 11°C for on-column digestion. The slurry was transferred into empty Proteus 1-Step Batch Mini Spin columns (Generon), and the mobile phase containing the protein was separated from the resin by centrifugation (500*g*, 5 min, 4°C). MBP-TEV was removed by batch binding for 30 min on 250 μl of amylose resin (New England Biolabs). Imidazole was removed by desalting using Zeba Columns 7K cutoff (Thermo Fisher Scientific). The protein concentration was determined by bicinchoninic acid (BCA) assay (Thermo Fisher Scientific).

### Thermostability measurements using differential scanning fluorimetry

Thermal unfolding analysis using dye-free differential scanning fluorimetry (nanoDSF) (*57*) was performed, as previously described (*13*). Approximately 6 μg of protein was added into a final volume of 50-μl buffer at the indicated pH values [40 mM bis-tris, 150 mM NaCl, TOCL (0.1 mg/ml), and 0.1% LMNG] in the presence or absence of sodium pyruvate. Samples (10 μl) were loaded into nanoDSF-grade standard glass capillaries and subjected to a temperature ramp (4°C per min from 25° to 95°C). The intrinsic fluorescence was measured in a Prometheus NT.48 nanoDSF device, and the apparent melting temperature (*T*_m_) was calculated from the inflection point using the PR.ThermControl software (NanoTemper Technologies).

### Thermostability shift assay using a cysteine-reactive probe

Inhibitor binding to wild-type human MPC1L/MPC2 or the indicated alanine mutants was assessed by thermostability shift assays using the cysteine reactive probe 7-diethylamino-3-(4′-maleimidylphenyl)-4-methylcoumarin (CPM). In total, 3 μg of purified MPC1L/MPC2 complex was incubated with the indicated concentrations of inhibitor or sodium pyruvate in assay buffer [20 mM bis-tris (pH 7.0), 150 mM NaCl, TOCL (0.1 mg/ml), and 0.1% LMNG] for 10 min on ice. In parallel, the CPM probe was diluted to 0.1 mg/ml in the same assay buffer and equilibrated for 10 min at room temperature in the dark before addition at 2 μg/ml to the protein sample (*58*, *59*) and further incubation for 10 min. The fluorescence intensity was measured using a rotory quantitative polymerase chain reaction (PCR) multi-sample instrument (Rotor-Gene Q, QIAGEN, The Netherlands) with a temperature ramp of 5.6°C per min over 25° to 90°C. The excitation and emission wavelengths were 460 and 510 nm, respectively. Apparent melting temperatures were determined with the Rotor-Gene Q software 2.3.

### Transport assays

The purified MPC1L/MPC2 complex was reconstituted in proteoliposomes on the basis of previously established procedures (*13*) with a different lipid composition. Specifically, *Escherichia coli* polar lipid extract, egg l-α-phosphatidylcholine 99% (w/v), and TOCL (all from Avanti Polar Lipids, AL, USA) were mixed in a 7.5:2.5:1 (w/w) ratio, dried under a stream of nitrogen, and hydrated in 20 mM tris-HCl (pH 8.0) and 50 mM NaCl to a concentration of 12 mg/ml, in the presence of 5 mM unlabeled pyruvate. Lipids were solubilized with 1.2% (v/v) pentaethylene glycol monodecyl ether (Merck), and purified protein was added at a lipid-to-protein ratio of 250:1 (w/w). Stepwise removal of pentaethylene glycol monodecyl ether was achieved by four additions at 20-min intervals of 60 mg of Bio-Beads SM-2 (Bio-Rad) and one 480-mg addition overnight with gentle mixing at 4°C. Proteoliposomes were separated from the Bio-Beads by passage through empty spin columns (Bio-Rad), pelleted at 120,000*g* for 60 min, and resuspended with a thin needle in 100 μl of their supernatant.

Pyruvate homo-exchange was measured at room temperature by 200-fold dilution of proteoliposomes in external buffer [20 mM MES (pH 6.4) and 50 mM NaCl] containing 50 μM [^14^C]-pyruvate (PerkinElmer). The reaction (0 to 30 s) was terminated by rapid dilution into eight volumes of ice-cold internal buffer [20 mM tris-HCl (pH 8.0) and 50 mM NaCl], followed by rapid filtration through cellulose nitrate 0.45-μm filters (MilliporeSigma) and washing with an additional eight volumes of buffer. The filters were dissolved in Ultima Gold scintillation liquid (PerkinElmer), and the radioactivity was counted with a PerkinElmer Tri-Carb 2800 RT liquid scintillation counter.

### Data analyses and representation

Statistical analyses were performed in GraphPad Prism version 10.2.3 (GraphPad Software, USA). A one-way analysis of variance (ANOVA) with Dunnett’s post hoc test for multiple comparisons was used to assess whether single alanine mutants were different from wild-type protein, while a one-way ANOVA with Tukey’s post hoc honest significance test was used to determine significant differences in substrate binding at different pH values. In all cases, *P* < 0.05 was considered significant.

Thermostability of substrate binding in certain alanine mutants occasionally required deconvolution of peaks to separate the unliganded from liganded protein populations, which was initially done in Fityk 3 (*60*) and confirmed in Prism. Initial rates of pyruvate transport were determined as previously described (*61*). IC_50_ measurements were determined as previously described (*14*).

### Generation of nanobodies targeting the MPC

The human MPC complex hMPC1L/MPC2 was reconstituted into l-α-phosphatidylcholine:TOCL (10:1 g/g ratio, Avanti Polar Lipids) at a 10:1 lipid:protein ratio in the absence of any ligand. These high-density proteoliposomes were immunized into one llama (*Lama glama*), after which a phage display library in the pMESy4 vector was prepared from peripheral blood lymphocytes using standard protocols (*62*). These procedures were carried out in strict compliance with the European legislation (EU directive 2010/63/EC) and the Belgian Royal Decree of 29 May 2013 regarding the protection of laboratory animals, and these animals were not specifically bred for such use. The llamas were taken care of in a farm licensed by the Belgian competent authorities (accreditation number LA 1700601) and provided with ample food, water, and room to roam. Four nanobody families were identified, containing six nanobodies in total.

Initial binding assays were performed with the hMPC1L/MPC2 complex to confirm interactions with the nanobodies. hMPC1L/MPC2 (75 μg) was incubated at a stoichiometric ratio with each nanobody for 1 hour at 4°C before addition of 100 μl of Ni–nitrilotriacetic acid (NTA) Superflow resin (QIAGEN) and a further 1-hour incubation at 4°C. This mixture was then centrifuged using Proteus 1-Step Batch Mini Spin columns (Generon), and the flowthrough was removed. The resin was washed in the column with 1 ml of buffer B, and, then, the protein was eluted with buffer B with 250 mM imidazole. The fractions (initial mixture, flowthrough, wash, and eluate) were separated on an SDS-PAGE gel.

### PMb generation and formation of the complex

For PMb construct generation, the sequence for nanobody CA19425 was amplified by PCR, using the pMESy4 plasmid as a template, and digested by KpnI and PvulI restriction enzymes (New England Biolabs). This was then ligated into the pBXNPHM3 vector (Addgene, no. 110099), so the nanobody could be expressed with an N-terminal MBP, cleavable with human rhinovirus 3C protease. PMb25 was then expressed and purified following previously described methods (*32*). *E. coli* MC1061 cells expressing PMb25 were cultured in 2 liters of terrific broth [supplemented with ampicillin (100 μg/ml), 0.1% glucose, 1 mM MgCl_2_, 1.0% glycerol, and 16 ml of supercharger buffer (3.48 M NH_4_Cl, 352 mM Na_2_SO_4_, and 1.21 M NaCl)] at 37°C. Once the optical density at 600 nm = 0.7, the cultures were induced with 0.02% arabinose for 3.5 hours, harvested, and resuspended in lysis buffer [150 mM NaCl, 50 mM tris-HCl (pH 8.0), 20 mM imidazole, 5 mM MgCl_2_, 10% glycerol, deoxyribonuclease I (10 μg/ml), and cOmplete Mini EDTA-free protease inhibitor tablet (Roche)]. After mechanical disruption (Constant Cell Disruption Systems) at 227.5 MPa, the cell debris was pelleted via centrifugation at 20,500*g* for 30 min at 4°C, and the supernatant was incubated with 3 ml of Ni-NTA slurry for 1 hour. After washing the resin with wash buffer [150 mM KCl, 40 mM imidazole (pH 7.5), and 10% glycerol], the protein was eluted with elution buffer [150 mM KCl, 300 mM imidazole (pH 7.5), and 10% glycerol] and incubated overnight with 3C protease (Merck). To remove the cleaved His-tagged N-terminal MBP, the sample was incubated the next day with 0.7 ml of Nickel Sepharose High Performance resin (GE Healthcare) for 1 hour. The flowthrough was collected and incubated with 750 μl of Strep-Tactin XT 4Flow slurry (IBA) for 1 hour. The resin was packed in a column and washed with 100 mM tris (pH 8.0), 150 mM NaCl, and 1 mM EDTA, and the PMb was eluted with 50 mM biotin. The final sample was passed through a PD-10 column for buffer exchange to buffer B [20 mM bis-tris (pH 7.0) and 150 mM NaCl]. After concentration using 10-kDa centrifugal filters (MilliporeSigma), the concentrations of both the purified PMb25 and the hMPC1L/2 heterodimer were determined by BCA assay (Thermo Fisher Scientific) and mixed at a 1:1.1 hMPC:PMb25 molar ratio. The hMPC/PMb25 complex was left to bind overnight at 4°C. The next morning, 40 μM C7, 250 μM zaprinast, or 250 μM mitoglitazone were added to the sample, in addition to 2 mM d-maltose and 0.035% fluorinated octyl maltoside, allowed to incubate for a further 2 hours, and then used for grid freezing.

### Sample preparation and cryo-EM data acquisition

Quantifoil R 1.2/1.3 300-mesh holey carbon copper grids, glow discharged for 30 s at 10 mA before sample freezing, were prepared and frozen with 3 μl of complex hMPC1L/MPC2 and PMb25 at 4°C, using an FEI Vitrobot IV operated at 4°C and under 95% humidity (Thermo Fisher Scientific) with blotting for 3.0 s at a blot force setting of −15. Screening images were taken using a Talos Arctica (Thermo Fisher Scientific) at 200 kV and ×73,000 magnification using a Falcon 3 detector in linear mode. The final datasets were collected from a single grid each using a Titan Krios (Thermo Fisher Scientific) at 300 kV at ×165,000 magnification equipped with a Falcon 4i detector and a Selectris X energy filter in counting mode. Data were acquired using EPU (version 3.1) at a size of 0.729 Å/pixel with defocus range of −1.8 to −2.4 μm in 0.2-μm increments and the autofocus routine run every 10 μm and with total exposure of 53.7 electrons/Å^2^ at 6.5 electrons/pixel per second. Data were acquired as four exposures per hole in aberration-free image shift mode with 5-s delay after stage shift and 1-s delay after image shift.

### Cryo-EM data processing

Data processing was performed using CryoSPARC v3.3.2. For each sample, ~8000 to 10,000 movies were collected, with the exception of the MPC-zaprinast sample, where two sessions were conducted, resulting in ~20,000 movies. Initial movie frame alignment, contrast transfer function parameter estimation, particle picking, and particle extraction (using a box size of 416 pixels) were carried out using Warp (*63*). The particles were then binned to a pixel size of 2.35 Å.The extracted particles were subjected to multiple rounds of two-dimensional classification to eliminate obvious artifacts, junks, and low-quality particles. Subsequently, an ab initio reconstruction in CryoSPARC was performed on 100,000 particles, which were divided into five or six classes. The resulting ab initio volumes, corresponding to different population of particles such as the MPC-macrobody complex, MPC without the macrobody, and empty micelle, were used as input for heterogeneous refinement. Two rounds of heterogeneous refinement were conducted to further segregate high-quality particles from potential contaminants. Last, nonuniform refinement was applied to the remaining particles, using the volume from the heterogeneous refinement as the reference, and particles were re-extracted to 0.729 Å/pixel. This process yielded a final reconstruction at a resolution of 3.40 to 3.97 Å.

For the MPC in the presence of mitoglitazone, a local refinement was performed following the final nonuniform refinement. The mask used for this job was generated using UCSF Chimera 1.13.1. The map was filtered with vop gaussian using a SD of 4. The threshold was carefully adjusted to encompass the carrier and the nanobody excluding the MBP.

### Model building, validation, and structural analysis

Model building was initiated using the cryo-EM map of C7-inhibited MPC. Visual inspection of the map revealed clear density for the transmembrane region of MPC and for the nanobody region of the PMb. Density for the amphipathic helices of MPC and for the MBP region of the PMb were less well resolved. AlphaFold (*64*, *65*) models of MPC2 (AF-O95563-F1-model_v4_MPC2.pdb) and MPC1 (AF-Q9Y5U8-F1-model_v4_MPC1.pdb), with side chains pruned to match the sequence of MPC1L using phenix.sculptor (*66*), were placed into the cryo-EM map using Chimera (*67*). The AlphaFold model of MPC1L (AF-P0DKB6-F1-model_v4_MPC1L.pdb) did not fit the experimental density well and was not used for model building. Model building for PMb25 was initiated using a homology model of a nanobody with high sequence identity [Protein Data Bank (PDB) code: 5IMM], generated by phenix.sculptor, and placed in the map using phenix.dock_in_map (*68*). Density for the inhibitor C7 was visible in the central cavity of MPC. Restraints for C7 were generated using the Grade Web Server (http://grade.globalphasing.org/) (*69*), and coordinates were fitted into the density using Coot (*70*) and Isolde (*71*). Iterative cycles of model rebuilding and real-space refinement were performed in Coot and Isolde, using secondary-structure restraints, against a 3.31-Å resolution density modified map produced by phenix.resolve_cryo_em (*72*).

Models for mitoglitazone-inhibited, zaprinast-inhibited, and apo-states of MPC were initiated by rigid body fitting the model of C7-inhibited MPC into the respective density maps using Chimera. Restraints for the inhibitors were generated using the Grade Web Server. For the apo-state, subsequent default flexible fitting in Isolde confirmed a significant conformational change to MPC. Iterative cycles of model rebuilding and real-space refinement were performed in Coot and Isolde, using secondary-structure restraints. Several factors guided modeling of the inhibitors: (i) stereochemistry, combined with minimal geometrical distortions following refinement, (ii) minimal clashes with protein residues, (ii) maximal number of protein-ligand interactions, and (iv) maximal cross-correlation with the cryo-EM map.

For each model, final positional minimization and group B-factor refinement was carried out in Servalcat and Refmac (*73*) against the CryoSPARC map. Over-fitting during refinement was monitored by shaking the coordinates of the model (0.3-Å RMSD), refining the shaken model against one of the half-maps (half-map A), and then calculating the FSC for the refined model against half-map A (FSC_work_) and for the refined model against the other half-map (FSC_test_). The final model of C7-inhibited MPC consists of residues 22 to 106 of MPC1L, residues 8 to 125 of MPC2, residues 4 to 131 of the PMb, and a molecule of C7. The final model of mitoglitazone-inhibited MPC consists of residues 6 to 106 of MPC1L, residues 8 to 123 of MPC2, residues 4 to 131 of the PMb, and a molecule of (*R*)- and (*S*)-mitoglitazone, each at 50% occupancy. The backbone of the amphipathic helix for MPC1L of mitoglitazone-inhibited MPC fits density in the map from nonuniform refinement, but side chains are not well-defined and are modeled as common rotamers. The final model of zaprinast-inhibited MPC consists of residues 15 to 105 of MPC1L, residues 8 to 121 of MPC2, residues 4 to 131 of the PMb, and a molecule of zaprinast. The final model of apo-inward MPC consists of residues 6 to 107 of MPC1L, residues 8 to 121 of MPC2, and residues 4 to 131 of the PMb.

The models were validated using Molprobity (*74*). The modeling of the inhibitors was not solely based on the cryo-EM maps but also considered chemical series, ligand geometry, clashes with protein residues, and maximal protein-inhibitor interactions. All possible binding modes were investigated independently by docking studies using Maestro (Schrödinger LLC, New York, NY, 2024) and SwissDock (*75*) and by molecular dynamics using the experimentally determined density features (Isolde) (*71*). In addition, we have carried out ligand docking studies with ChemEm (*76*), a program optimized for ligand docking into cryo-EM maps, which supports our models. Protein-ligand interactions and protein-protein interactions were analyzed using LigPlot+ and Dimplot (*77*) and the Protein-Ligand Interaction Profiler web server (*78*). Electrostatic surfaces were generated by Adaptive Poisson–Boltzmann Solver (APBS) (*79*) and visualized in PyMOL (*80*). The mitoglitazone-inhibited and apo-inward structures were rigid body aligned by jCE (*81*) before analyzing conformational changes using the DynDom server (https://dyndom.cmp.uea.ac.uk/dyndom/) (*82*). Protein interface surface areas were calculated using PDBePISA (*83*). Protein structure figures were prepared in PyMOL (*80*). In some figures, the Zappo color scheme was used to indicate their properties, where positively charged, negatively charged, polar, aliphatic, aromatic, Gly/Pro, and Cys residues are colored blue, red, green, pink, orange, magenta, and yellow, respectively.

### Structural modeling of human MPC complexes

Structural models were built using AlphaFold-Multimer v2.2.0 (*64*, *84*). Sequences of MPC protomers were taken from the UniProt database: MPC1_human (UniProt ID: Q9Y5U8), MPC1L_human (UniProt ID: P0DKB6), and MPC2_human (UniProt ID: O95563). For the MPC1L_human sequence, the C-terminal residues D116-S136 were truncated because they had very low confidence [low predicted Local Distance Difference Test (pLDDT) scores].

Two sets of structures were built, with 100,000 structures per set: MPC1/MPC2 heterodimer and MPC1L/MPC2 heterodimer. We varied the depth of the multiple sequence alignment used to build the models to sample different conformational states. We used 10 different settings: default settings, reduced Big Fantastic Database (BFD); no metagenomics databases (removed BFD and only used two sequences from MGnify); no metagenomics databases; and Uniref90 depth capped at 2500, 2000, 1500, 1000, 500, and 50 sequences. We built 10,000 structural models per setting, except for the setting with no metagenomics databases and Uniref90 depth capped at 1500 sequences, for which we built 20,000 structural models.

### Analysis of MPC structural models determined by AlphaFold

The quality of the dimer was assessed with ProQDock score (*85*); models with a score greater than 0.4 were retained for further analysis. This score has been trained to reproduce the fold protein/protein interfaces; values above 0.4 represent medium-quality interactions. To assess the closure of the transporter on each side of the membrane, we used a metric that counts, with a smooth function, how many atoms of each of the pathways are within a cutoff. This coordination number is defined as

$$C=\sum_{i\in \text{group}1}\sum_{j\in \text{group}2}\frac{1-{\left(r_{\mathrm{ij}}/R\right)}^{6}}{1-{\left(r_{\mathrm{ij}}/R\right)}^{12}}$$

where *i* and *j* refer to a non-hydrogen atom in each group, *r*_*ij*_ is the distance between these atoms in a given structural model, and *R* is 4.5 Å. The greater the number and proximity of contacts formed between the two groups of atoms, the higher the coordination number.

Gating coordination number metrics were computed to define closure on the N-terminal and C-terminal sides of the protein. For the N-terminal gating metric, the following groups of residues were used: (i) 66, 69, 73, 74, 77, and 80 in MPC1, or 68, 71, 75, 76, 79, and 82 in MPC1L; and (ii) 82, 85, 89, 90, 93, and 96 in MPC2. For the C-terminal gating metric, the following groups of residues were used: (i) 45 to 53 in MPC1 or 47 to 55 in MPC1L; and (ii) 61 to 69 in MPC2. These variables were computed using PLUMED library, version 2.2 (*86*–*88*). For example, a coordination number value for the N-terminal gating of ~100 will indicate that around 100 atoms of the N-terminal side are in close proximity, and, therefore, the pathway is closed.

The structural models were clustered in the space of these C- and N-gating coordination numbers using the *k*-medoids clustering algorithm (*89*) from the METAGUI 3 tool (*90*). The number of clusters was 9, and the maximum distance matrix dimension was 5000.

### Docking of pyruvate to MPC1L/MPC2 occluded representative model

The protein and ligand were protonated and minimized using Maestro (Schrödinger LLC, New York, NY, 2024). We assessed the three possible protonation states of the histidine-86 in the binding site. Pyruvate docking poses were sampled using the InducedFit docking methodology of Maestro (Schrödinger LLC, New York, NY, 2024) (*91*) within a box centered at residues H86 of MPC1L and 49, 64, and 100 of MPC2, the size of which was determined automatically; the number of poses generated was set to 80.

The docking poses were clustered on the basis of the non-hydrogen atoms of pyruvate and the binding site residues using the conformer clustering module of Maestro (Schrödinger LLC, New York, NY, 2024). We used the average linkage clustering method without fitting, and the cluster number was at a minimum of the Kelley function (*92*). The resultant clusters were analyzed according to cluster size and docking scores.
