# Supplementary material for: Molecular basis of pyruvate transport and inhibition of the human mitochondrial pyruvate carrier
Source: Sci Adv. 2025 Apr 18;11(16):eadw1489. doi: 10.1126/sciadv.adw1489 (PMC12007569; doi:10.1126/sciadv.adw1489)
Supplement: Supplementary file 1 — Figs. S1 to S24 Tables S1 and S2 Legends for movies S1 and S2 [file sciadv.adw1489_sm.pdf]

Science Advances
AAAS

Supplementary Materials for

Molecular basis of pyruvate transport and inhibition of the human mitochondrial pyruvate carrier

Maximilian Sichrovsky *et al.*

Corresponding author: Edmund R. S. Kunji, ersk2@cam.ac.uk; Vanessa Leone, vleone@mcw.edu

Sci. Adv. **11**, eadw1489 (2025)
DOI: 10.1126/sciadv.adw1489

The PDF file includes:

Figs. S1 to S24
Tables S1 and S2
Legends for movies S1 and S2

Other Supplementary Material for this manuscript includes the following:

Movies S1 and S2

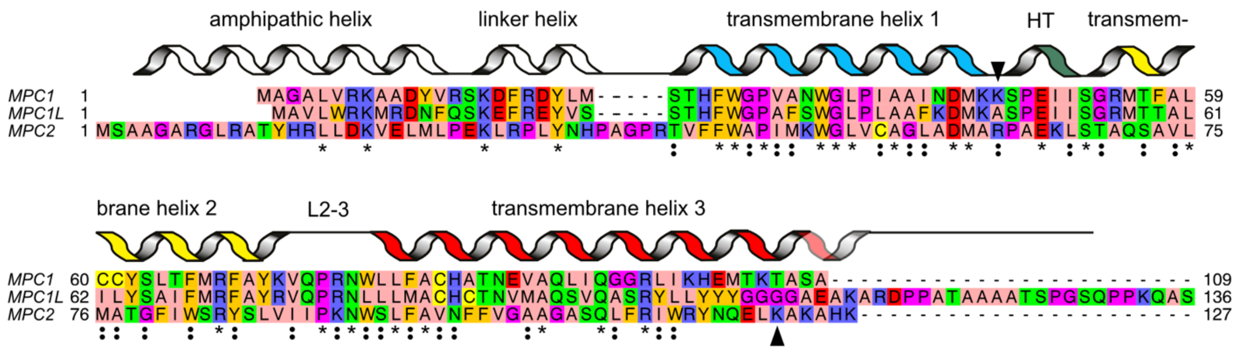

Fig. S1. Sequences and secondary structures of MPC protomers

Sequence alignment and secondary structural elements of human MPC1, MPC1L and MPC2, shown in the ZAPPO color scheme, where positively charged, negatively charged, polar, aliphatic, aromatic, Gly/Pro, and Cys amino acid residues are colored blue, red, green, pink, orange, magenta and yellow, respectively. Identical and similar residues in all three sequences are indicated with an asterisk and colon, respectively. The arrow heads indicate the lysine residues that are labeled with the membrane impermeable sulfo-NHS acetate in submitochondrial particles, which are inside-out compared to the topology in mitochondria.

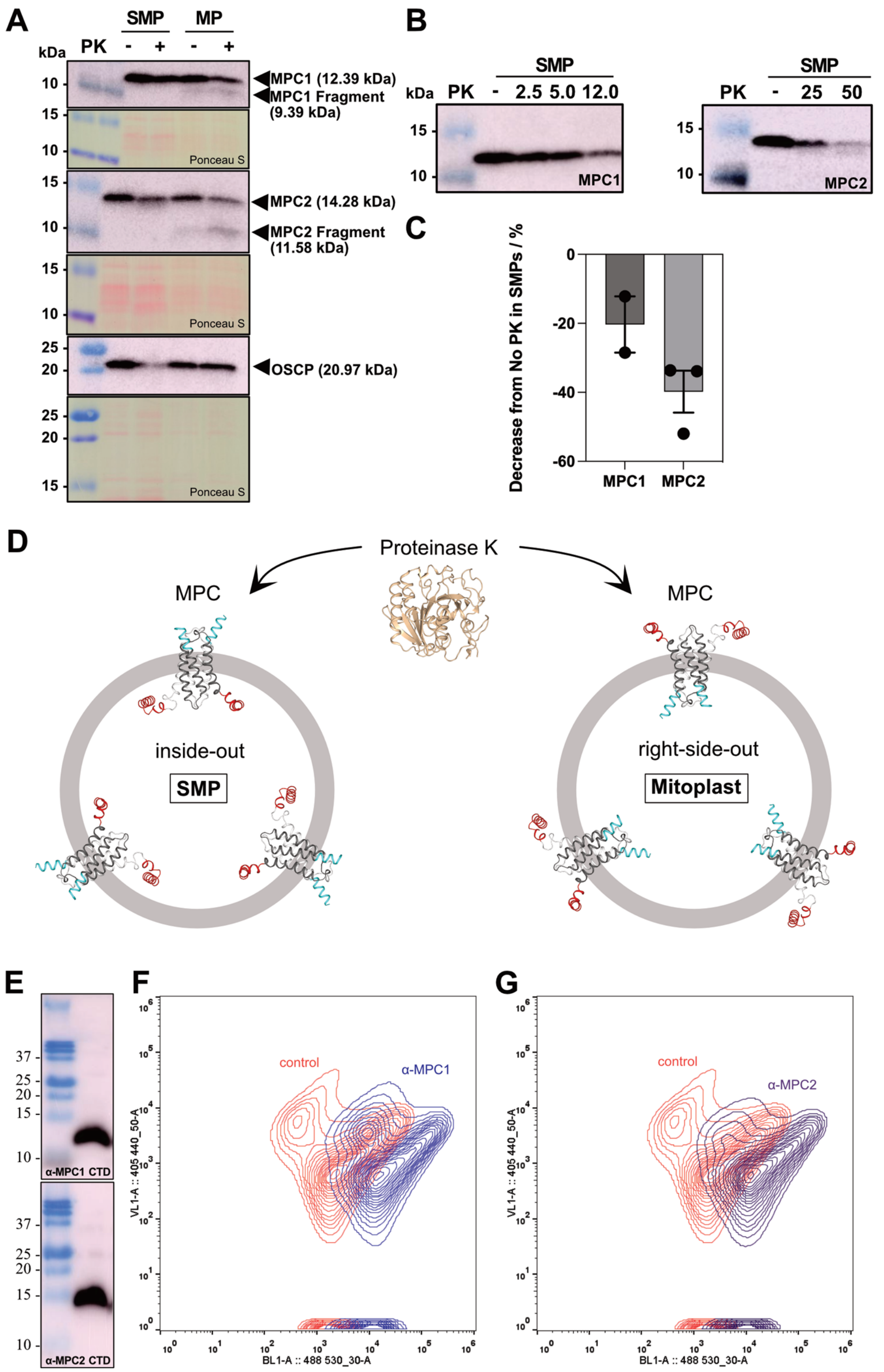

Fig. S2 Orientation of MPC by limited proteolysis, labeling, antibody binding experiments
(A) Limited proteolysis assay in right-side-out mitoplasts and inside-out submitochondrial particles (SMPs), prepared from bovine heart mitochondria. The samples were treated for 1 min with 2.5 µg/mL proteinase K (PK) for MPC1 samples and for 60 min with 25 µg/mL PK for MPC2 and OSCP samples. The samples were analyzed by SDS-PAGE and Western Blotting using antibodies against the C-termini of MPC1 and MPC2, and against the OSCP subunit of ATP

synthase. (B) Limited proteolysis assay in SMPs prepared from bovine heart mitochondria. The samples were treated with increasing concentrations of PK, as indicated, and analyzed by Western blotting. (C) The signal from Western blots in (B) was quantified with Fiji and normalized against the amount of total protein, as defined by Ponceau staining. Normalized data is shown as an average of two (MPC1) or three (MPC2) biological repeats of proteinase treatment at 2.5 µg/mL (MPC1) or 25 µg/mL (MPC2). (D) Diagram showing the determined topology and orientation of bovine MPC in mitoplasts and SMPs. The N-terminal amphipathic helices and the C-terminal antibody epitopes are shown in cyan and red, respectively. (E) Western blot of SMPs, showing the specificity of the peptide chicken antibodies against MPC1 (top) and MPC2 (bottom). (F-G) Flow cytometry analysis, showing (F) the binding of α-MPC1 (blue, left panel) and (G) α-MPC2 (purple, right panel) antibodies to SMPs compared to the no-secondary antibody control (red), using Alexa Fluor 488–conjugated goat anti-Chicken IgY (H+L) secondary antibodies. Contour plots represent fluorescence intensity measured in two channels: BL1-A (488/530 nm) and VL1-A (405/440 nm). Increased fluorescence in the BL1-A channel for both α-MPC1 and α-MPC2 conditions indicates specific antibody binding to MPC1 and MPC2, respectively, showing that the C-terminus is accessible in SMPs.

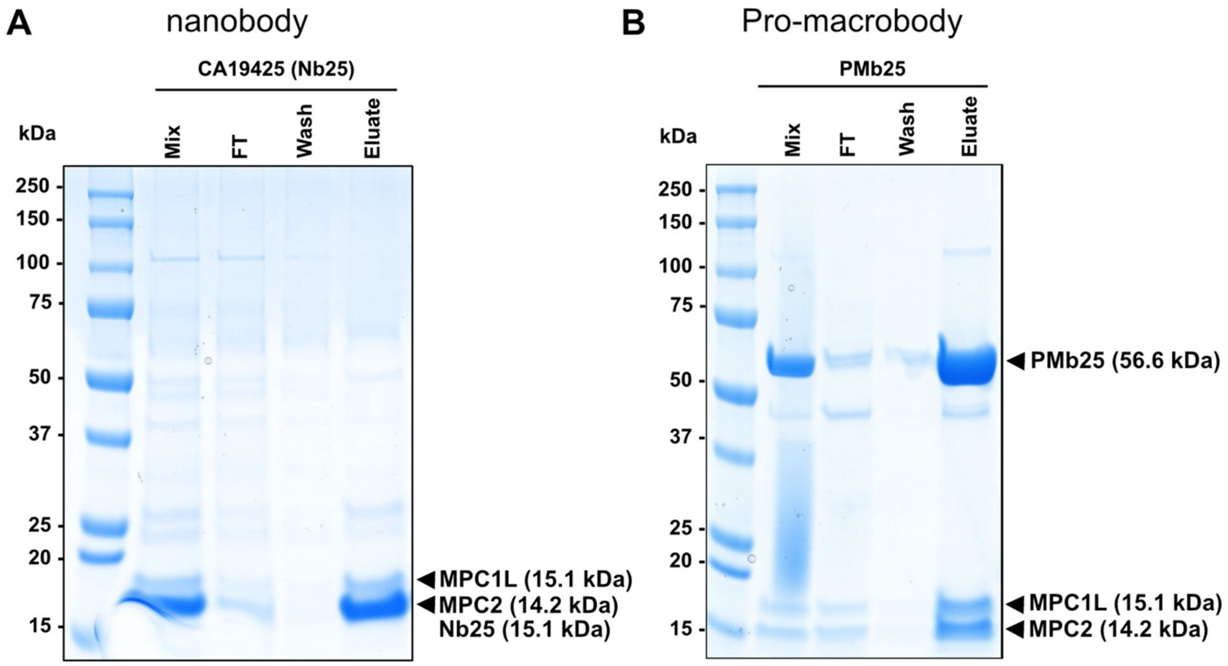

Fig. S3. Nanobody and Pro-macrobody binding studies.

Fractions from initial binding assays are shown for (A) Nb25 and (B) PMb25, showing binding to the human MPC1L-MPC2 complex. The fractions (initial mixture, flow-through (FT), wash, and eluate) were separated on a 12-20% SDS-PAGE gel with the eluate fractions showing bands

corresponding to MPC1L (15.1 kDa), MPC2 (14.2 kDa), Nb25 (15.1 kDa) and PMb25 (56.6 kDa), as indicated by arrow heads. Nb25 and MPC2 have approximately the same weight.

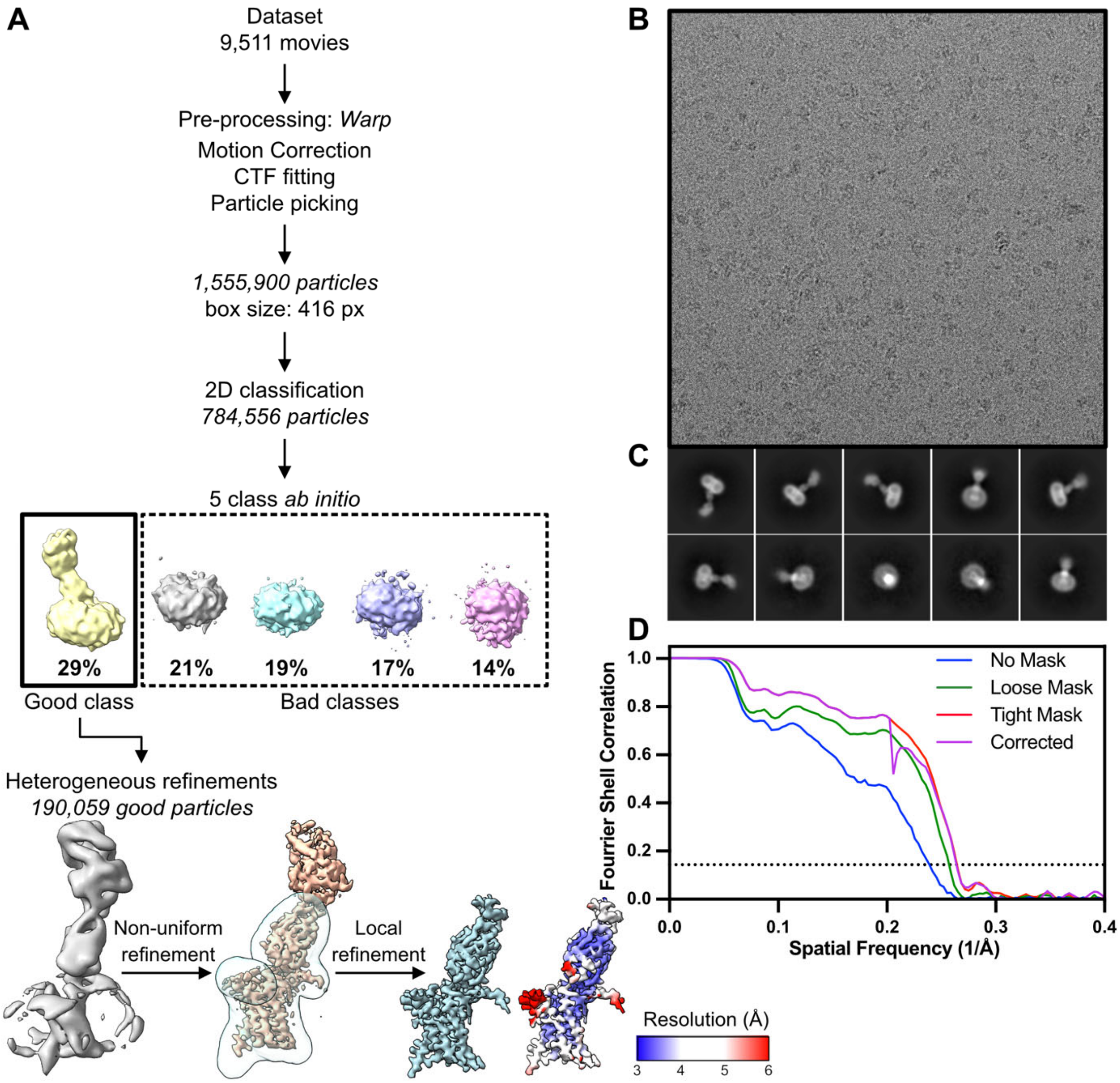

Fig. S4. Diagram of the dataset processing path of mitoglitazone-inhibited MPC in the outward-open state

(A) Flow chart of data processing in CryoSPARC v3.3.2. (B) Representative micrograph and (C) 2D class averages. (D) FSC curves as calculated by CryoSPARC v3.3.2, dotted line represents FSC = 0.143.

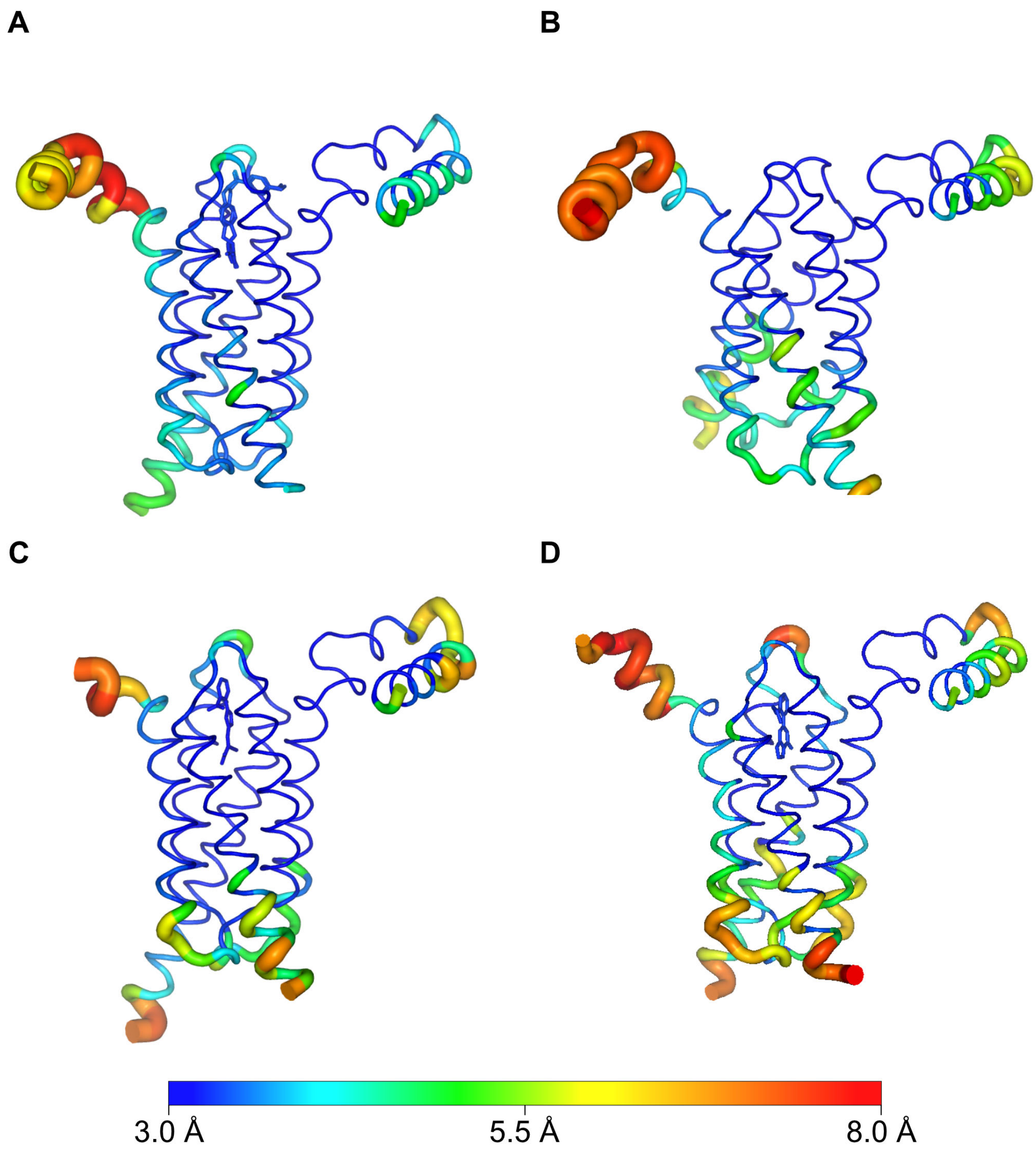

Fig. S5 Local map resolution at modeled atom positions of the mitochondrial pyruvate carrier

The local map resolution is shown for (A) mitoglitazone-inhibited outward-open state, (B) apo inward-open state, (C) C7-inhibited outward-open state, and (D) zaprinast-inhibited outward-open state of MPC. Each structure is shown as a cartoon in putty representation and colored by the local resolution, as shown in the scale-bar.

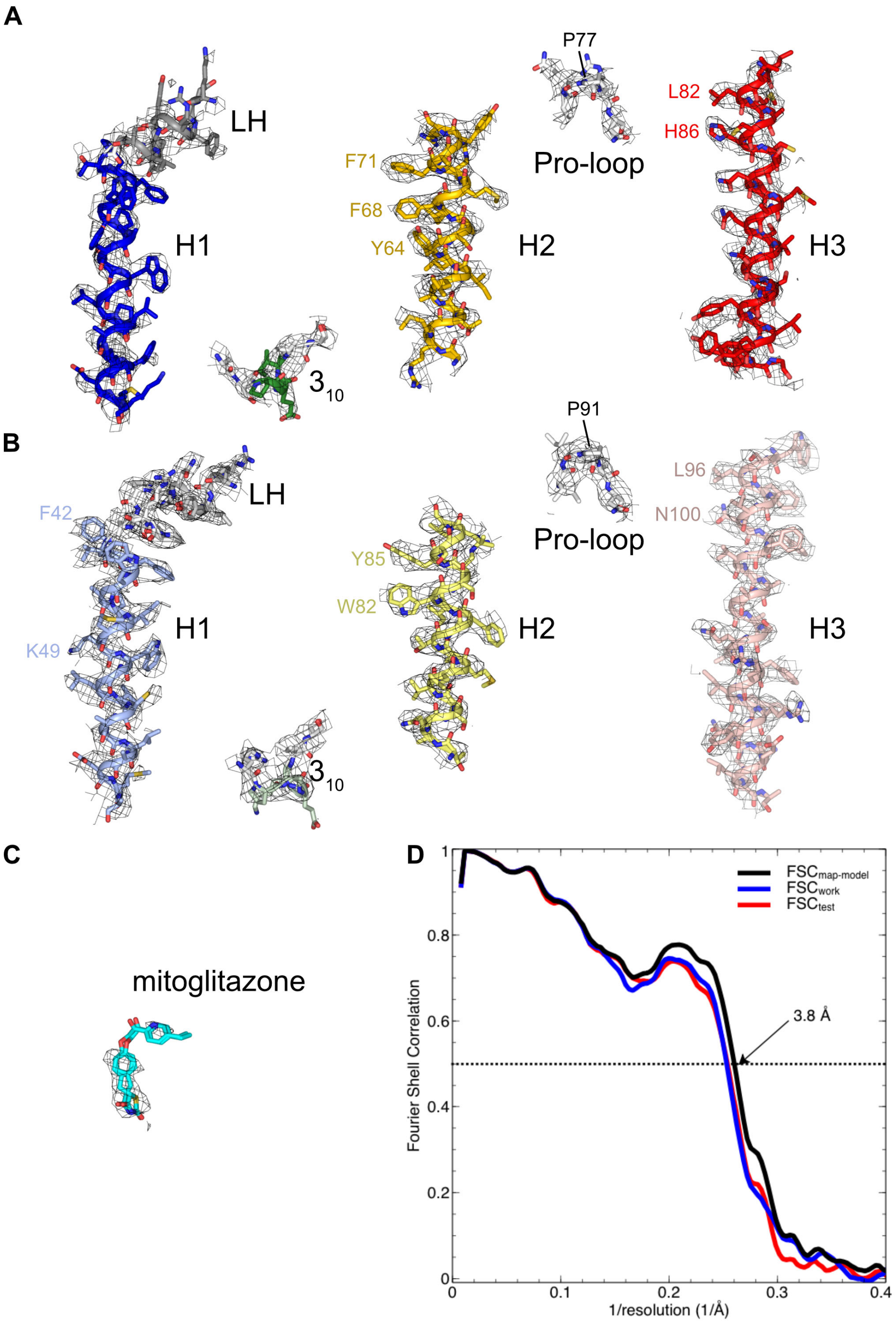

Fig. S6. Cryo-EM map quality and model validation for mitoglitazone-inhibited MPC in the outward-open state.

Transmembrane and linker helices, and connecting loops, of (A) MPC1L and (B) MPC2, with side chains shown in stick representation, and key residues indicated. (C) MTG shown in stick representation. Density maps in (A)-(C) are contoured at a Chimera contour level of 0.4 (9.1 σ) and shown as a black mesh within 2 Å of the depicted feature. (D) FSC of the refined model against

the map (black curve) plotted alongside FSCwork (blue curve) and FSCtest (red curve) validation curves.

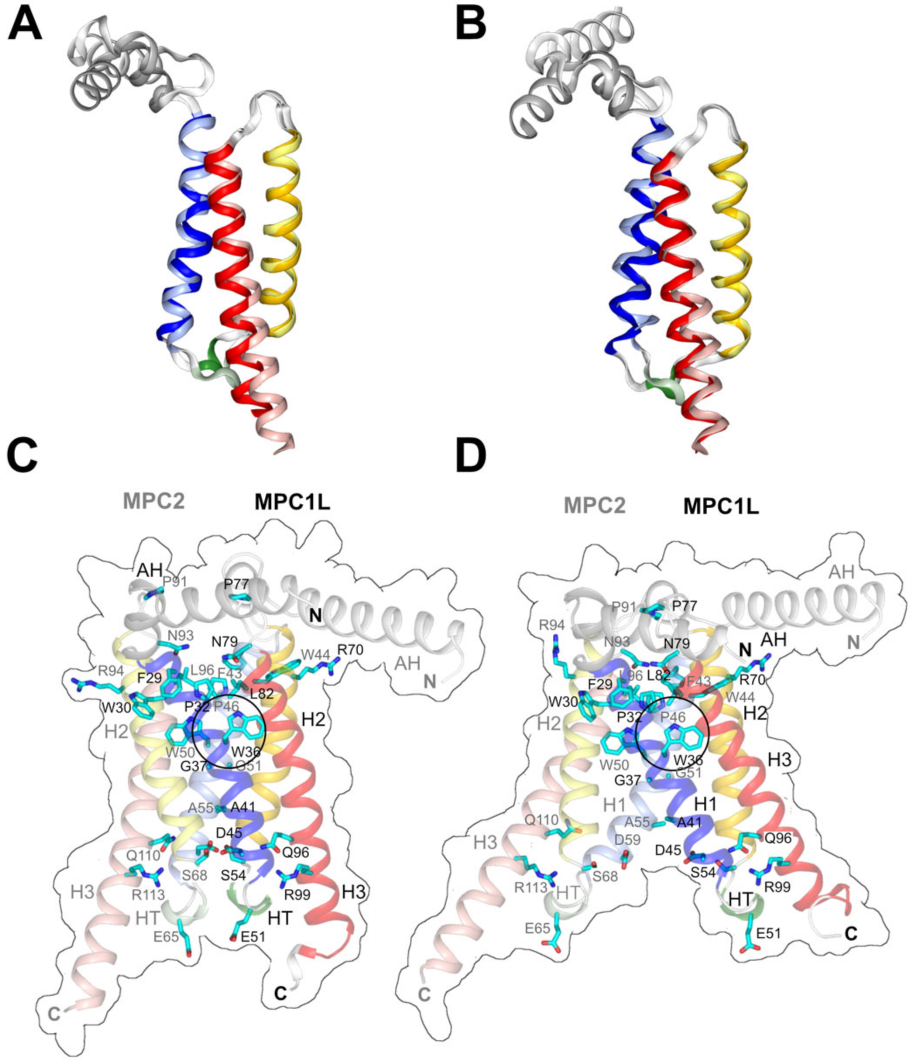

Fig. S7 Structural two-fold pseudo-symmetry and sequence conservation of symmetry-related residues of the mitochondrial pyruvate carrier. Structural alignment of the MPC1L and MPC2 domains in the (A) outward-open and (B) inward-open state. The conserved symmetry-related residues in the (C) outward-open and (D) inward-open state are shown cyan sticks and their residue numbers. Amphipathic helices (AH) and linker helices (LH) are colored gray, transmembrane helix 1 (H1) blue, helix 2 (H2) yellow, helix 3 (H3) red, and the 3_{10}-helix green. The surface is indicated. Lighter colors and labels are used for MPC2.

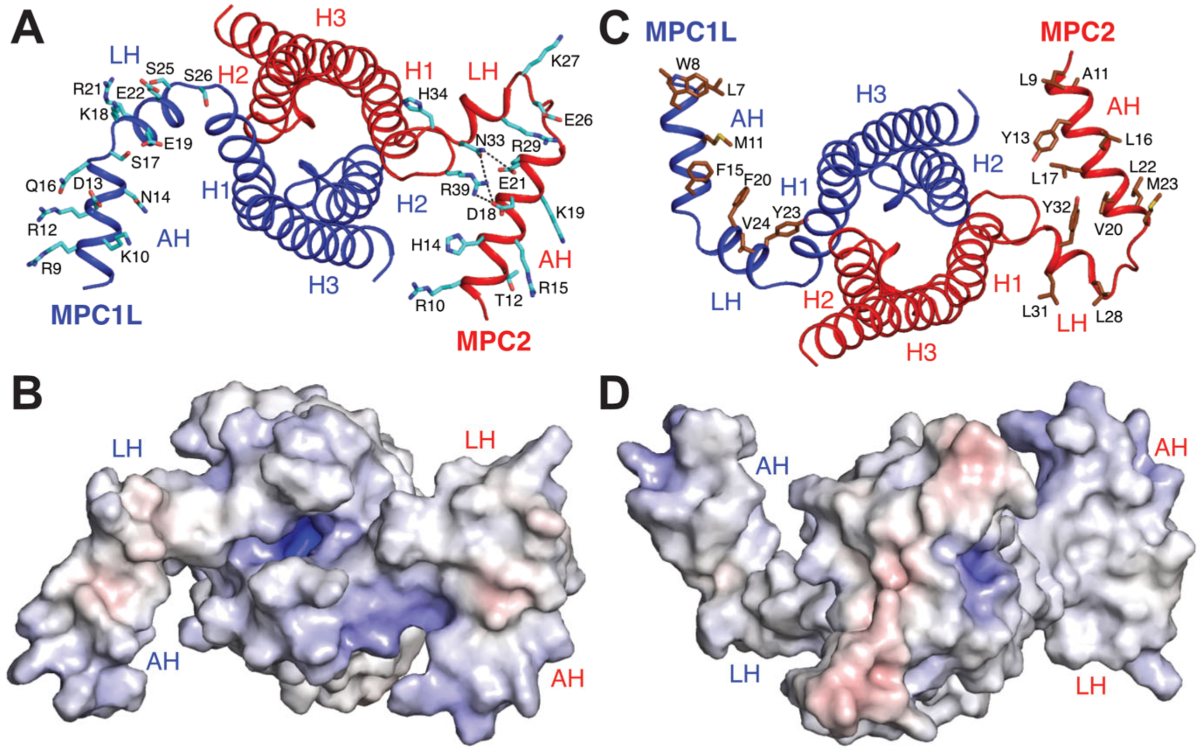

Fig. S8. Properties of the linker and amphipathic helices of the mitochondrial pyruvate carrier. (A) the polar and charged residues (cyan), (B) the electrostatic surface facing the water phase of the intermembrane space, (C) the hydrophobic residues (brown), and (D) electrostatic surface facing the hydrophobic core of the membrane. (A and C) MPC1L and MPC2 are shown in blue and red and polar and hydrophobic residues are shown in cyan and brown stick representations, respectively. (B and D) The surface is colored by electrostatic potential, as in Fig. 1. (A) The interactions between the polar D18, E21, N33 and R39, causing a helix-turn-helix in MPC2, are shown as black dashes.

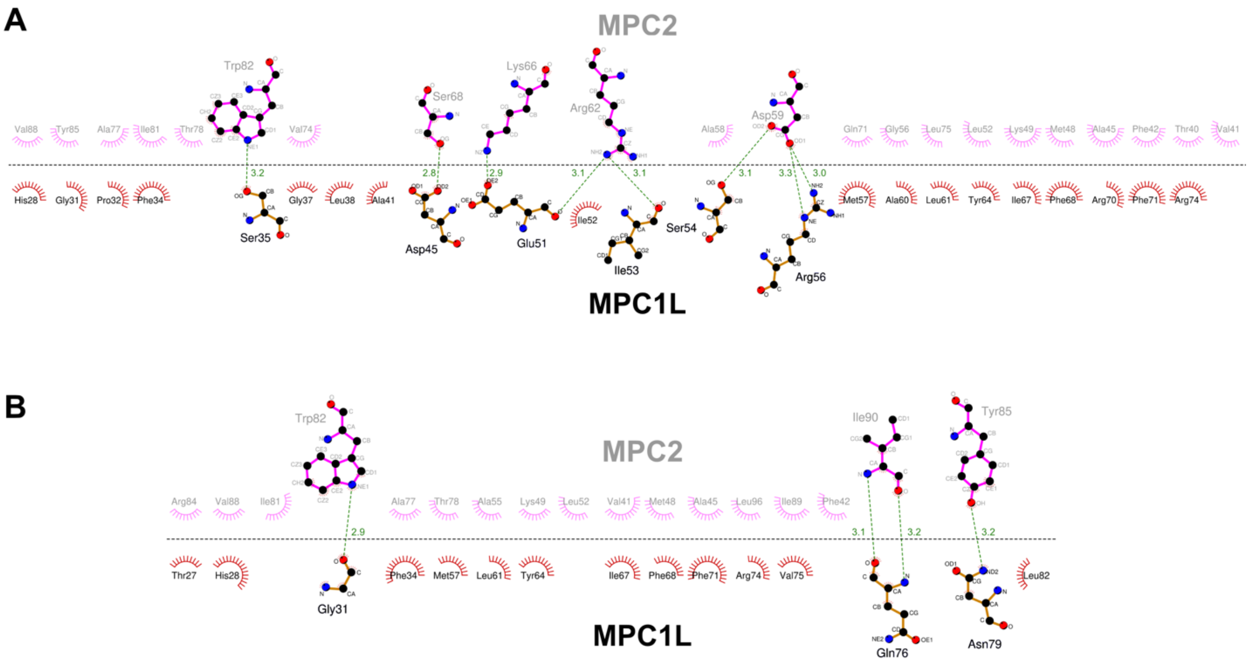

Fig. S9. Interactions between subunits of MPC in two states. Schematic drawing of the interface between MPC1L (residues labeled in black) and MPC2 (residues labeled in gray) of (A) mitoglitazone-inhibited outward-open state and (B) apo inward-open state. Amino acid residues within 4 Å of residues in the other subunit are shown. Hydrogen bonds and salt bridges are shown as green dashed lines with indicated distances (Å). Red arcs indicate residues in hydrophobic contact, with spokes radiating toward the atoms they contact. Figure generated by Dimplot.

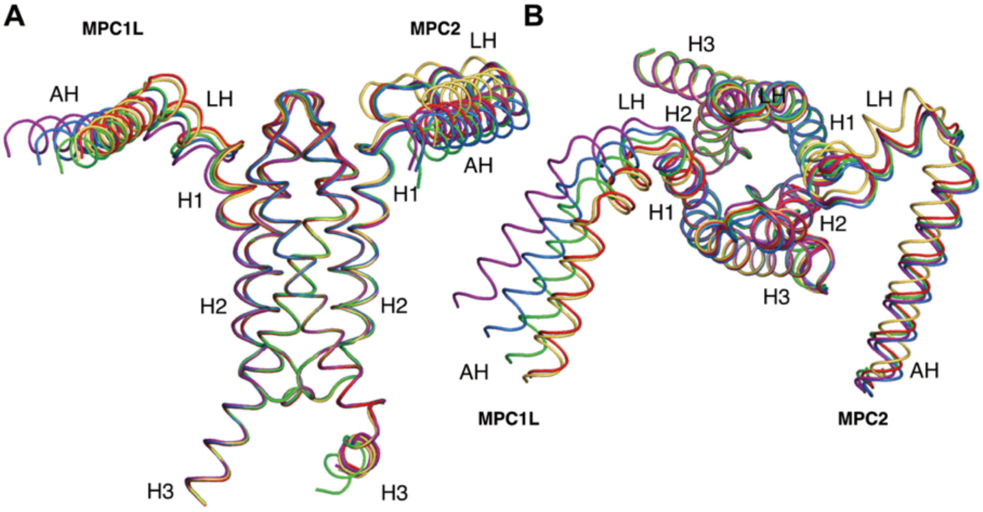

Fig. S10. Alignment of five closely related AlphaFold 2 models of MPC, representing the same outward-open state. (A) Lateral (left) and cytoplasmic views (right) of the structure. Amphipathic helices (AH), linker helices (LH) and transmembrane helix 1 (H1), helix 2 (H2) yellow, and helix 3 (H3) are indicated.

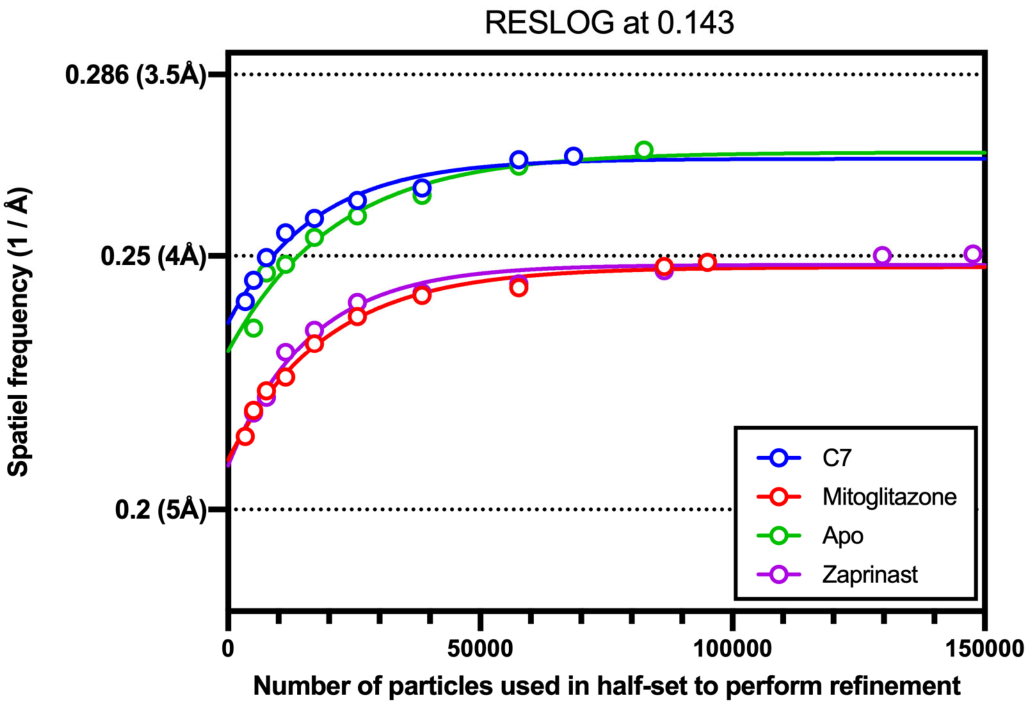

Fig. S11. Plot of the number of particles against the spatial frequency. For the four datasets in the paper, we have made the assessment whether further data collection would improve the resolution by using the ResLog analysis of CryoSPARC.

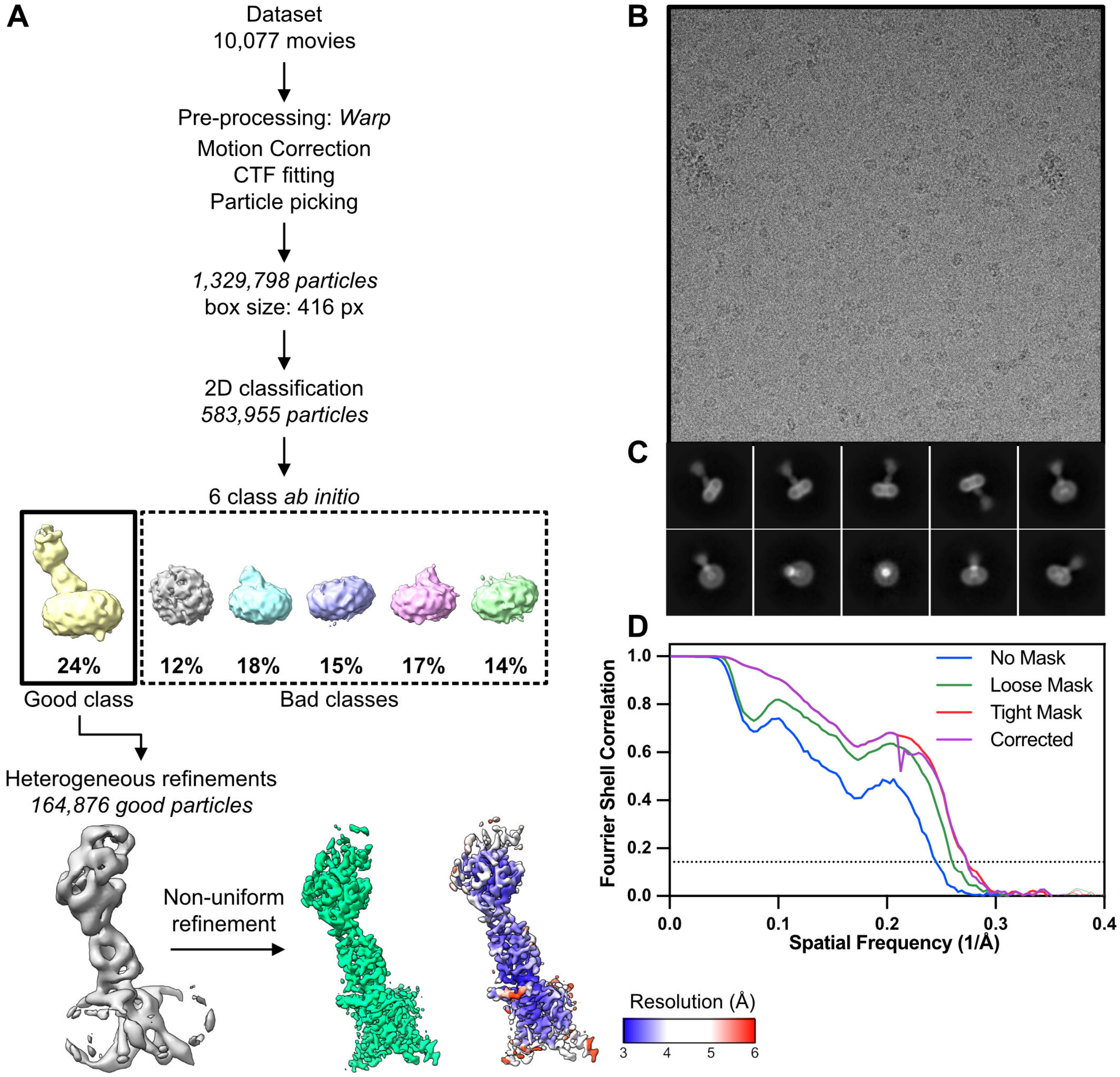

Fig. S12. Diagram of the dataset processing path of apo MPC in the inward-open state. (A) Flow chart of data processing in CryoSPARC v3.3.2. (B) Representative micrograph and (C) 2D class averages. (D) FSC curves as calculated by CryoSPARC v3.3.2, dotted line represents FSC = 0.143.

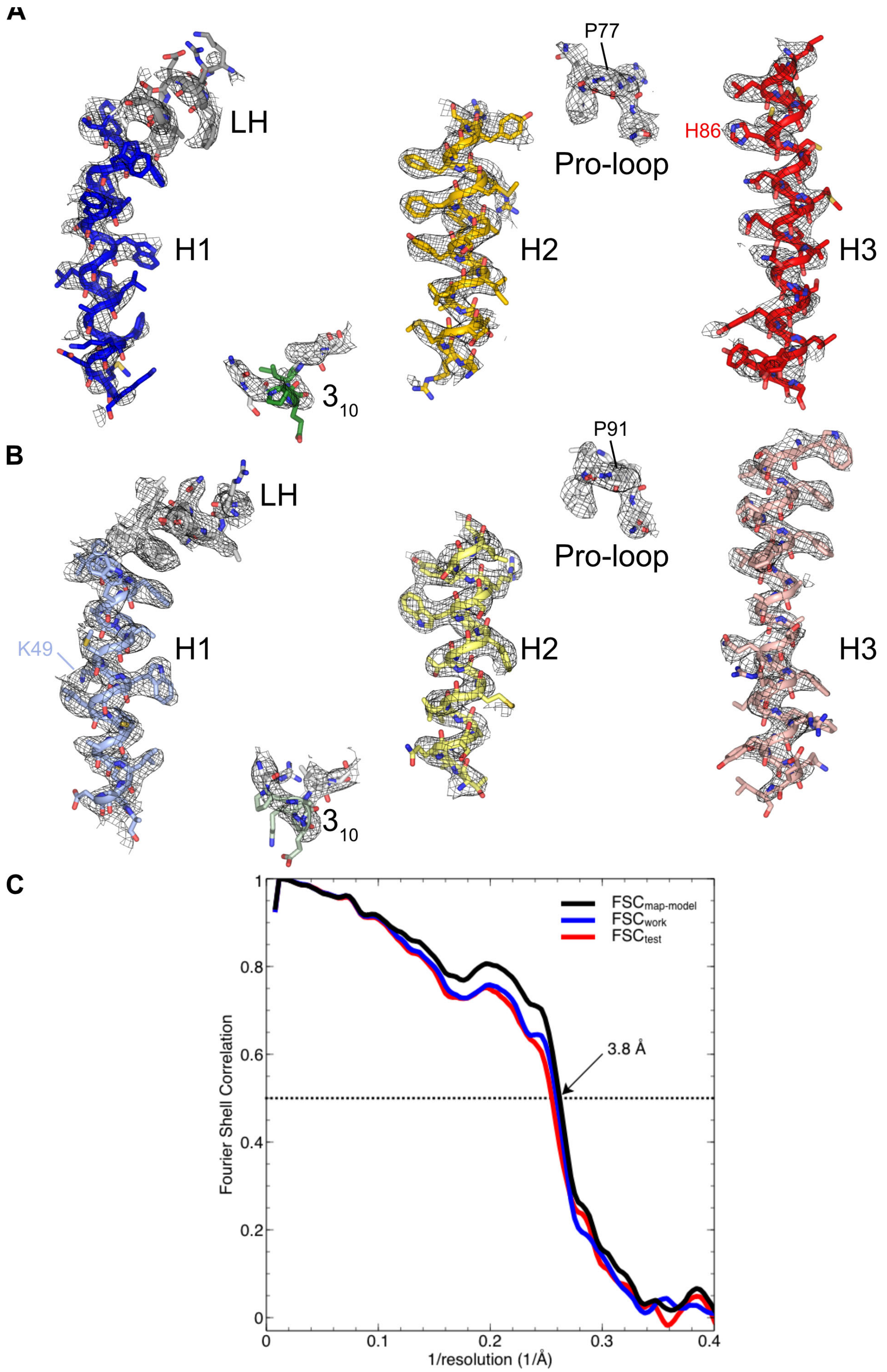

Fig. S13. Cryo-EM map quality and model validation for MPC in the inward-open state. Transmembrane and linker helices, and connecting loops, of (A) MPC1L and (B) MPC2, with side chains shown in stick representation. Density maps in (A)-(B) are contoured at a Chimera contour

level of 0.14 (10.9 σ) and shown as a black mesh within 2 Å of the depicted feature. (D) FSC of the refined model against the map (black curve) plotted alongside FSC_{work} (blue curve) and FSC_{test} (red curve) validation curves.

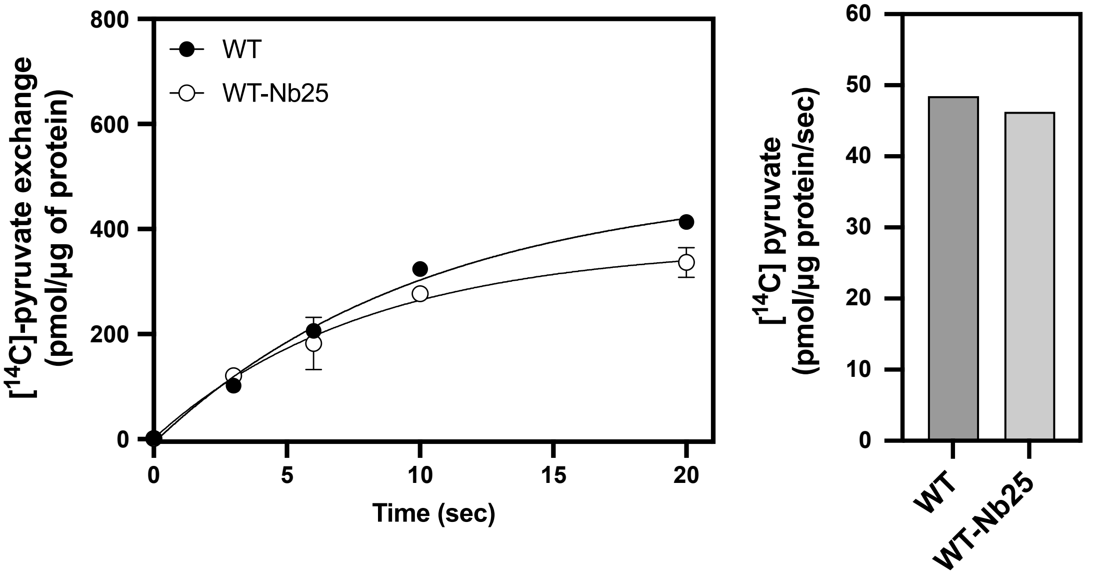

Fig. S14. Pyruvate transport by MPC1L/2 with and without bound nanobody 25.

(A) Time course of pyruvate homo-exchange at a ΔpH of 1.6 for MPC1L/2 reconstituted into proteoliposomes, with and without the addition of Nb25 prior to reconstitution. (B) Initial rates of transport for the two conditions.

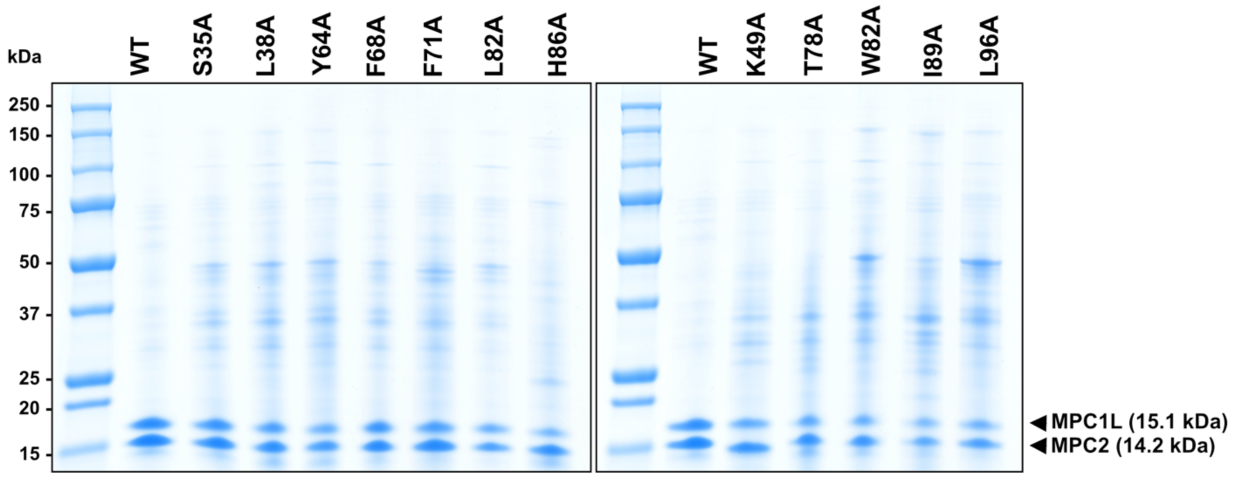

Fig. S15. Engineering of single alanine replacement mutants of **MPC1L-MPC2.**

Purified MPC heterodimers with single alanine replacements, showing MPC1L mutants (left panel) and MPC2 mutants (right panel). The proteins were separated on a 12-20% SDS-PAGE gel and stained with Coomassie.

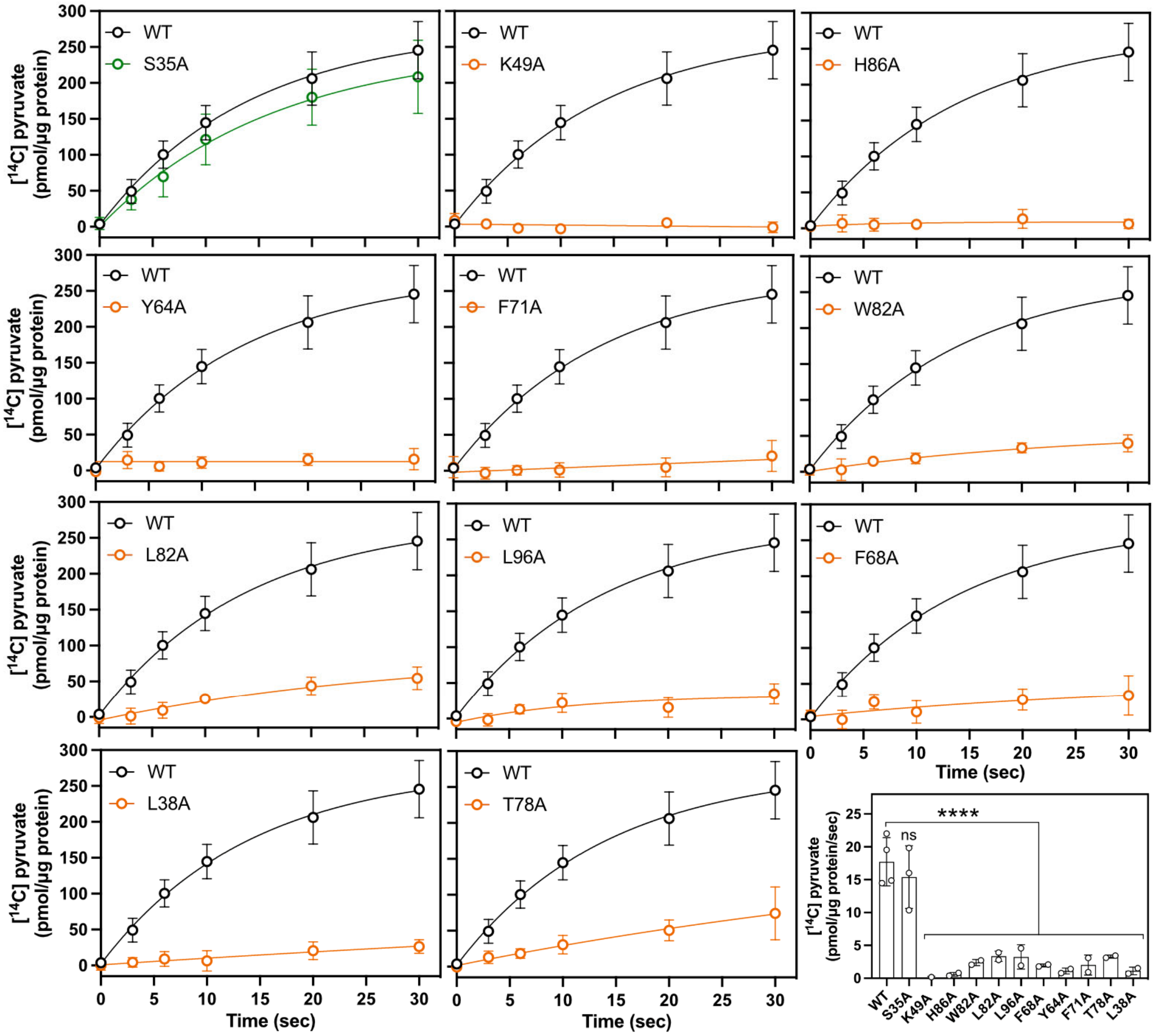

Fig. S16. Pyruvate transport by single alanine replacement mutants of residues in the binding pocket.

Time course of pyruvate homo-exchange at a ΔpH of 1.6 for alanine mutants reconstituted into liposomes. Values represent the mean and standard deviation of biological repeats, six for wild type, three for S35A and two for the rest of the mutants. Bottom right panel shows the initial rates of transport and the statistically significant differences between wild-type and mutants (ns = $p>0.05$, ***$p<0.001$).

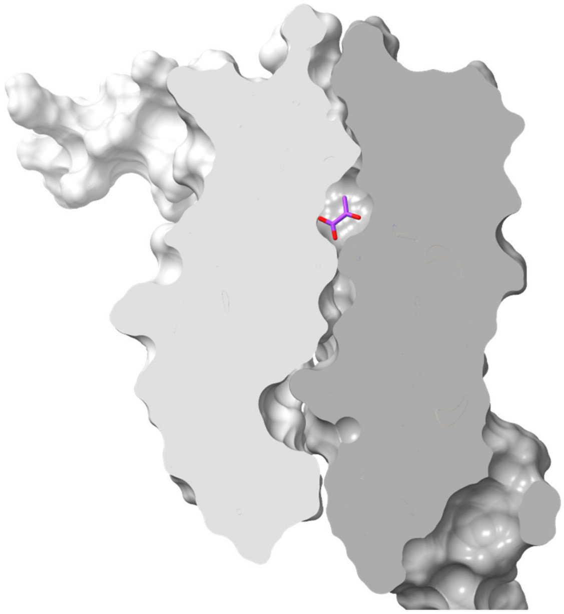

Fig. S17. Pyruvate binding pocket in the occluded state. Surface cutout of the representative occluded model of MPC1L (light grey)/MPC2(dark grey) upon pyruvate docking with H86 charged.

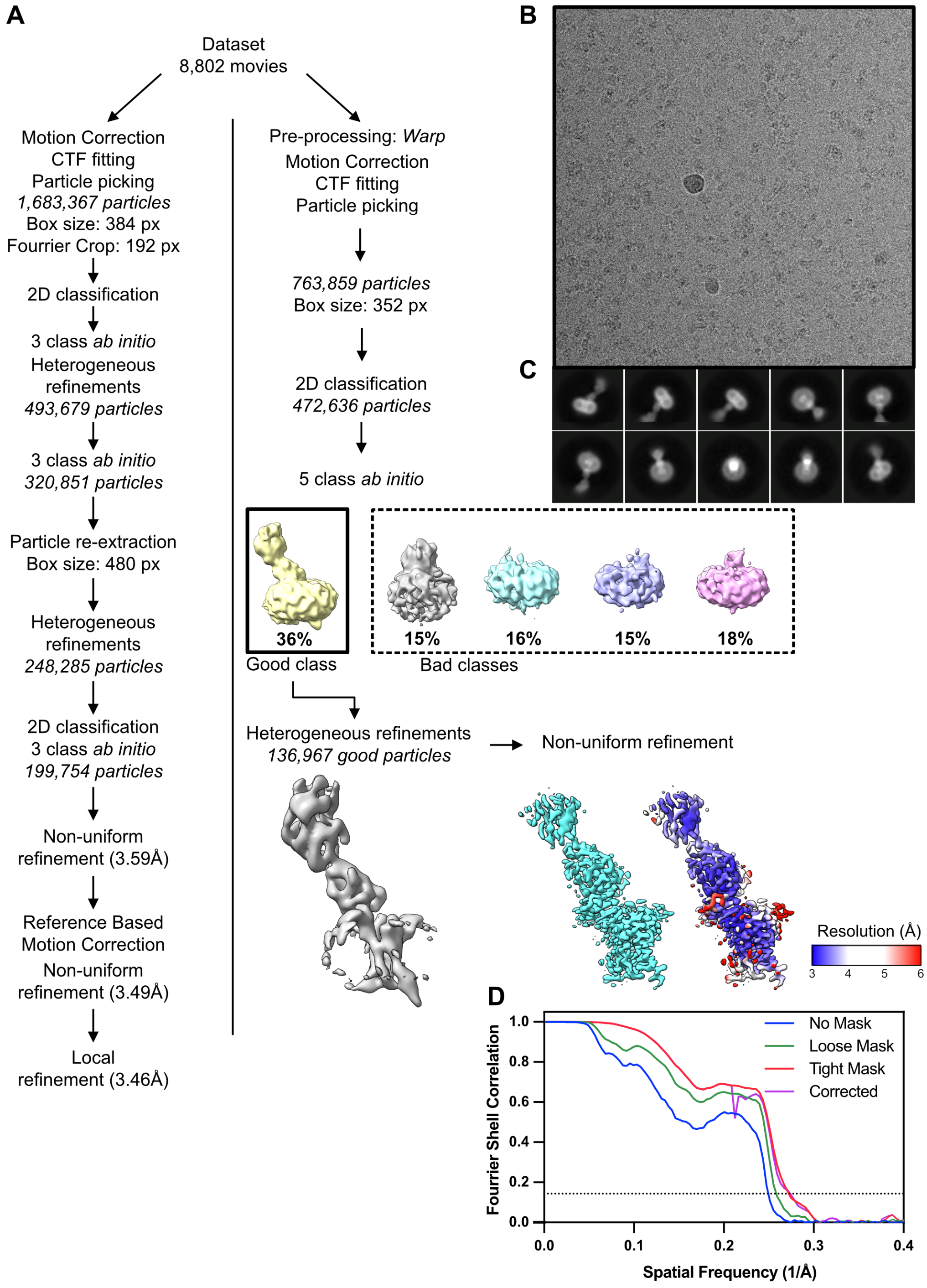

Fig. S18. Diagram of the dataset processing path of compound 7-inhibited MPC. (A) Flow chart of data processing in CryoSPARC v4.5.3 (left) and v3.3.2 (right). (B) Representative micrograph and (C) 2D class averages. (D) FSC curves as calculated by CryoSPARC v3.3.2, dotted line represents FSC = 0.143.

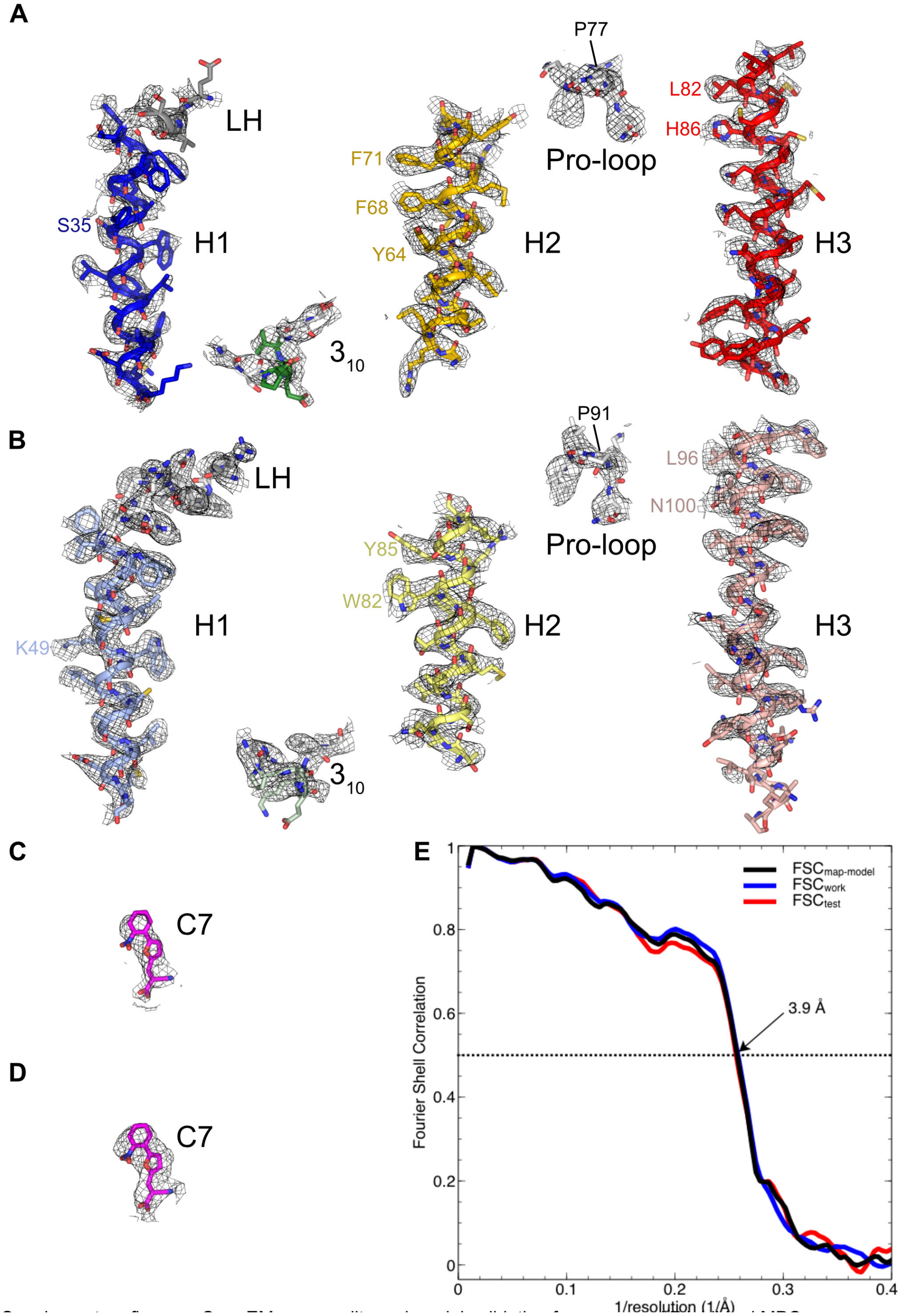

Fig. S19. Cryo-EM map quality and model validation for compound 7-inhibited MPC Transmembrane and linker helices, and connecting loops, of (A) MPC1L and (B) MPC2, with side chains shown in stick representation, and key residues labelled. (C) Compound 7 shown in stick representation. Density maps in (A)-(C) are from CryoSPARC non-uniform refinement, are contoured at a Chimera contour level of 0.14 (8.3$\boldsymbol{\sigma}$) and shown as a black mesh within 2 Å of the depicted feature. (D) Density-modified map in the region of compound 7, contoured at 1$\boldsymbol{\sigma}$. (E)

FSC of the refined model against the CryoSPARC map (black curve) plotted alongside FSC_{work} (blue curve) and FSC_{test} (red curve) validation curves.

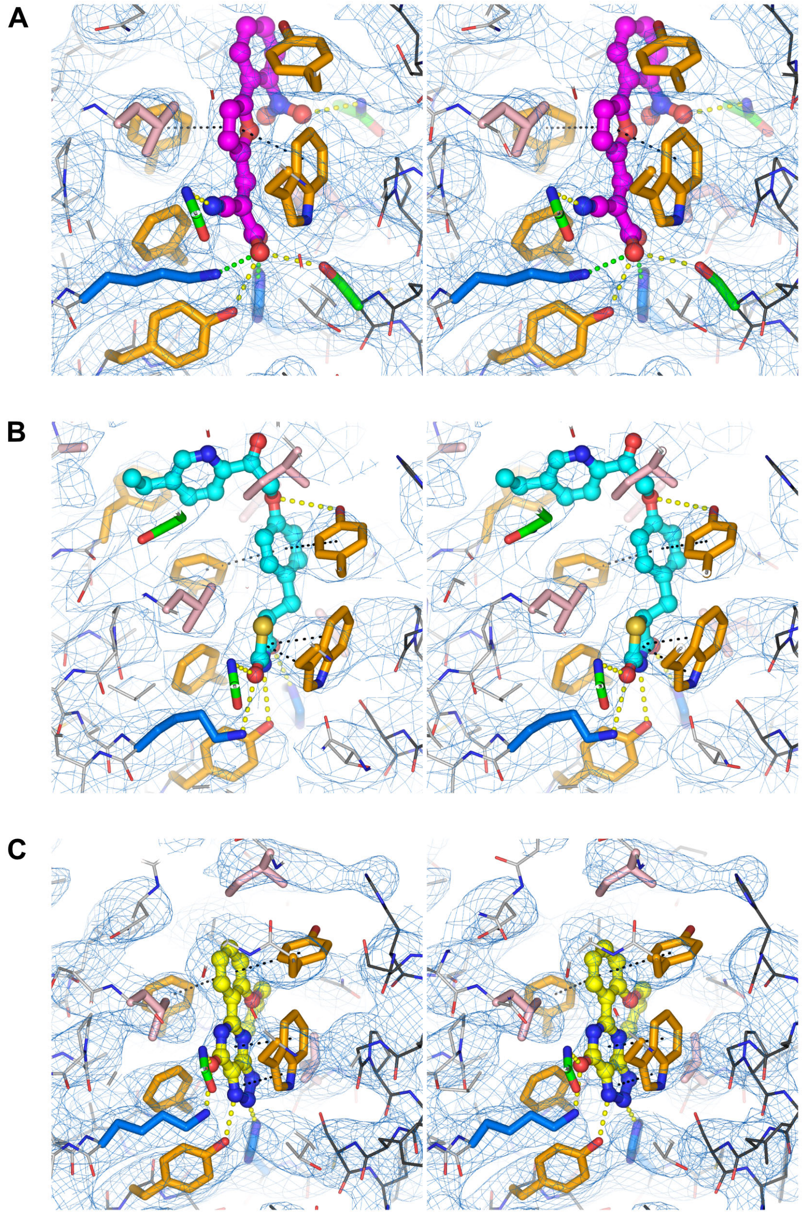

Fig S20: Wall-eyed stereo views of the cryo-EM maps around the inhibitor-binding sites (A) compound 7, (B) mitoglitazone and (C) zaprinast. Inhibitors and binding-site residues are shown in stick representation, viewed and colored as in panel (C) of figs. 4-6. Additional residues of MPC1L and MPC2 are shown as thin lines, with carbons colored dark gray and light gray, respectively. Cryo-EM maps are contoured at Chimera contour levels of (A) 0.14 (8.3 σ), (B) 0.4 (9.1 σ) and (C) 0.13 (10.4 σ), and shown as a blue mesh within 2 Å of the depicted feature. The mean local resolutions are 3.2 Å for compound 7, 3.5 Å for mitoglitazone, and 3.4 Å for zaprinast.

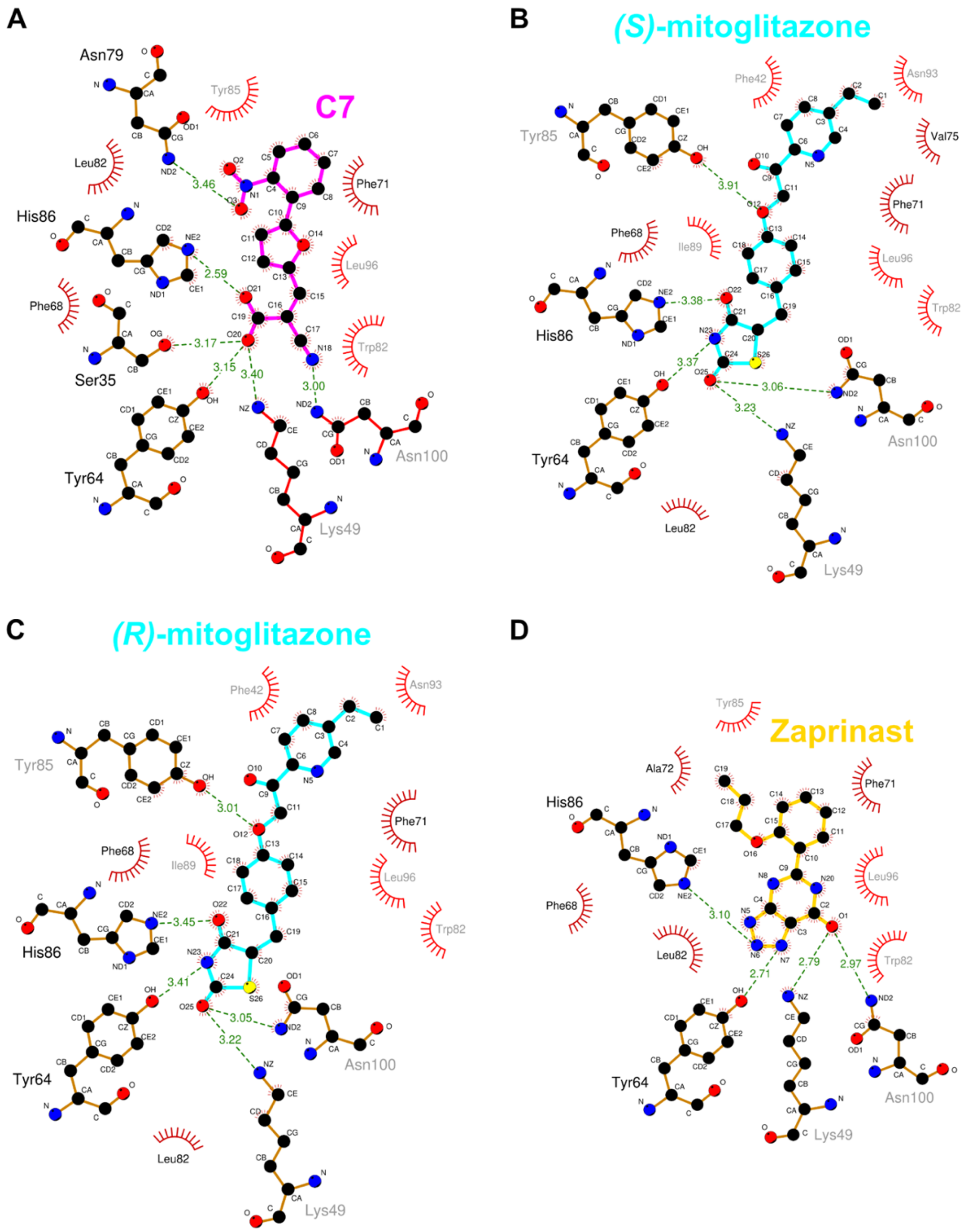

Fig. S21. Interactions of the inhibitors with residues of MPC.

Schematic diagrams of the binding site of (A) compound 7, (B) (*S*)-mitoglitazone, (C) (*R*)-mitoglitazone and (D) zaprinast. Amino acid residues within 4 Å of the inhibitor are depicted, with MPC1L residues labeled in black and MPC2 residues in gray. Hydrogen bonds and salt bridges are shown as green dashed lines with indicated distances (Å). Red arcs indicate residues in hydrophobic contact with the inhibitor, with spokes radiating toward the atoms they contact. Figure generated by Ligplot+.

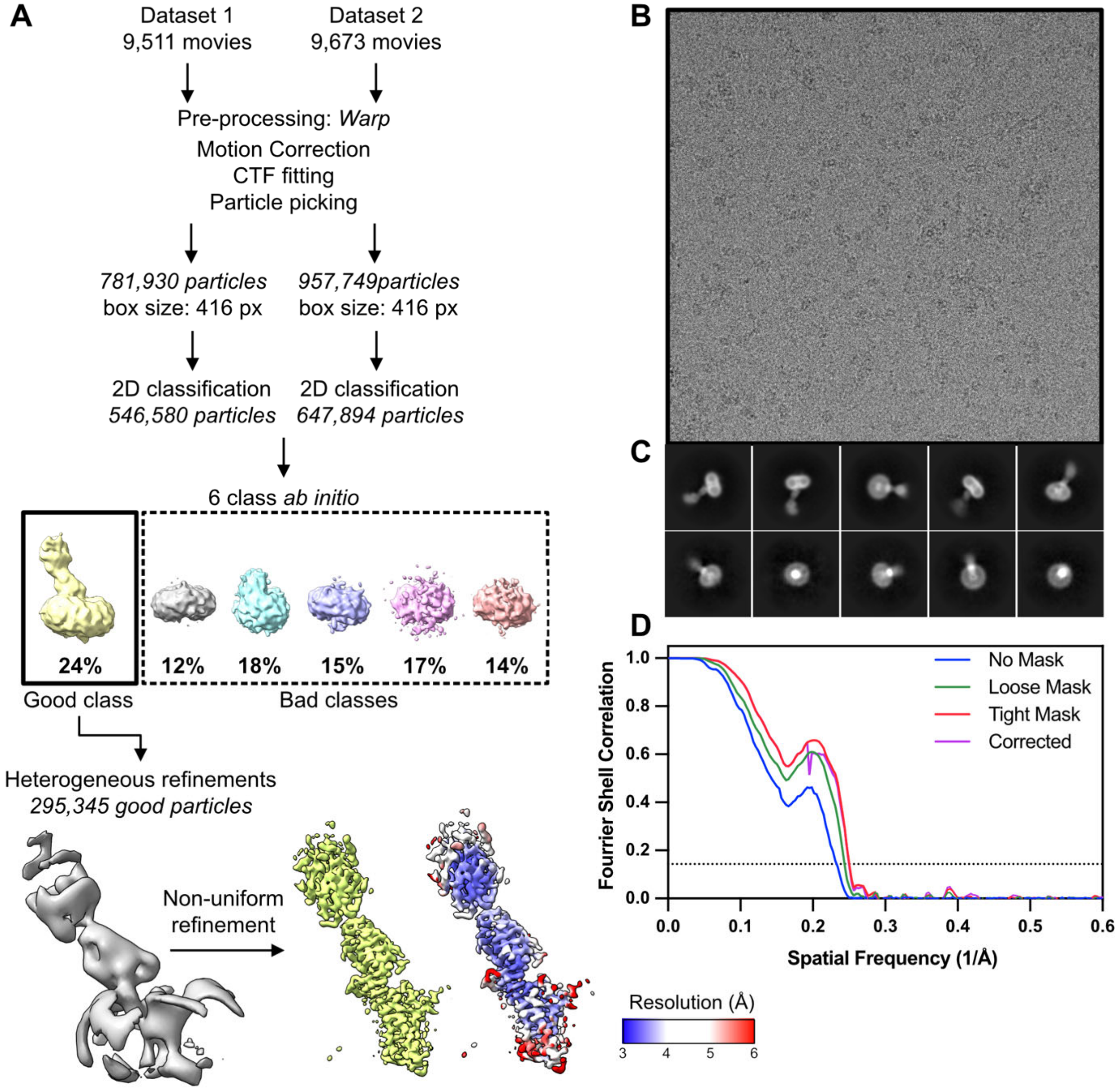

Fig. S22. Diagram of the dataset processing path of zaprinast-bound MPC. (A) Flow chart of data processing in CryoSPARC v3.3.2. (B) Representative micrograph and (C) 2D class averages. (D) FSC curves as calculated by CryoSPARC v3.3.2, dotted line represents FSC = 0.143.

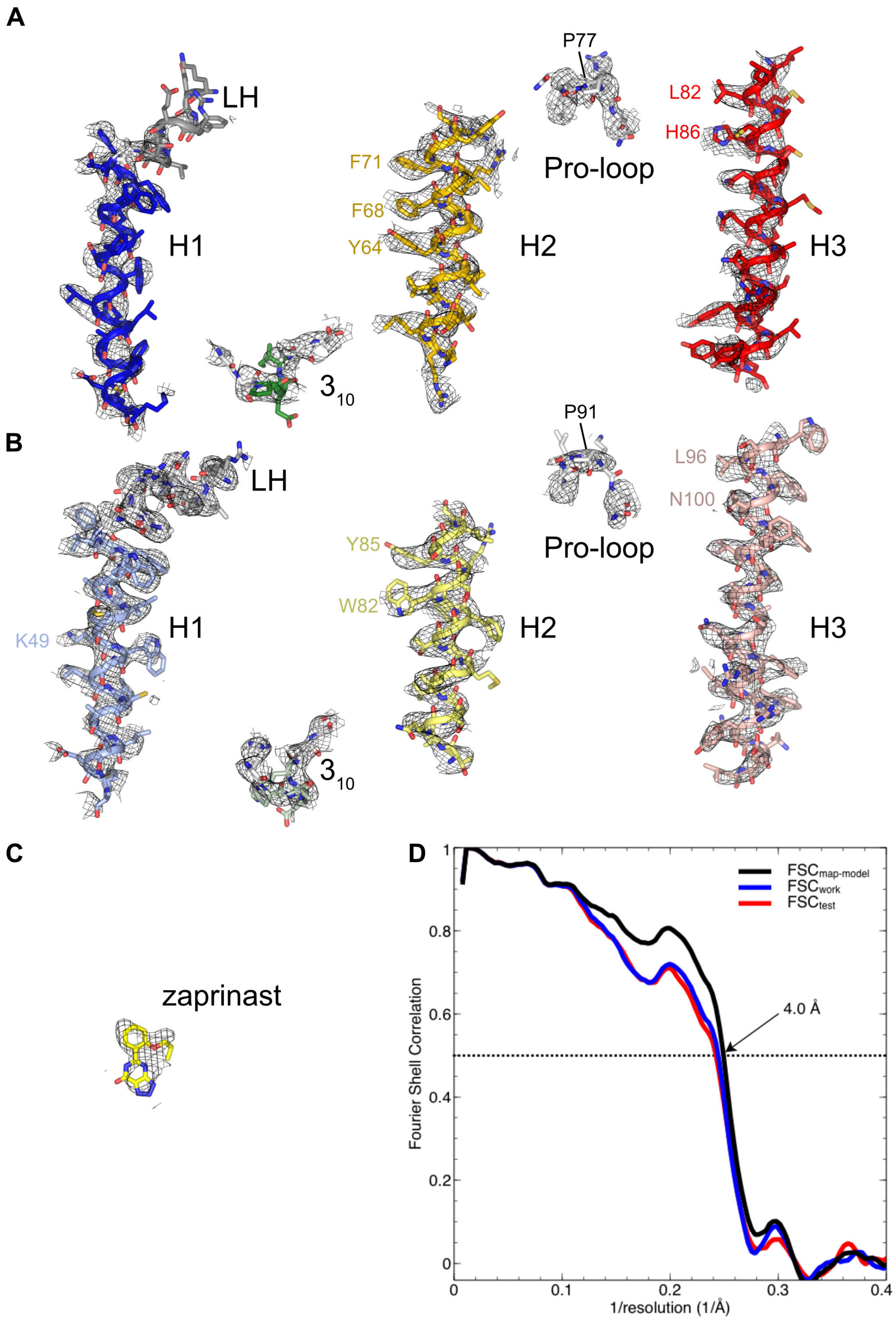

Fig. S23. Cryo-EM map quality and model validation for zaprinast-inhibited MPC. Transmembrane and linker helices, and connecting loops, of (A) MPC1L and (B) MPC2, with side chains shown in stick representation, and key residues labelled. (C) Zaprinast shown in stick

representation. Density maps in (A)-(C) are contoured at a Chimera contour level of 0.13 (10.4 σ) and shown as a black mesh within 2 Å of the depicted feature. (D) FSC of the refined model against the map (black curve) plotted alongside FSC_{work} (blue curve) and FSC_{test} (red curve) validation curves.

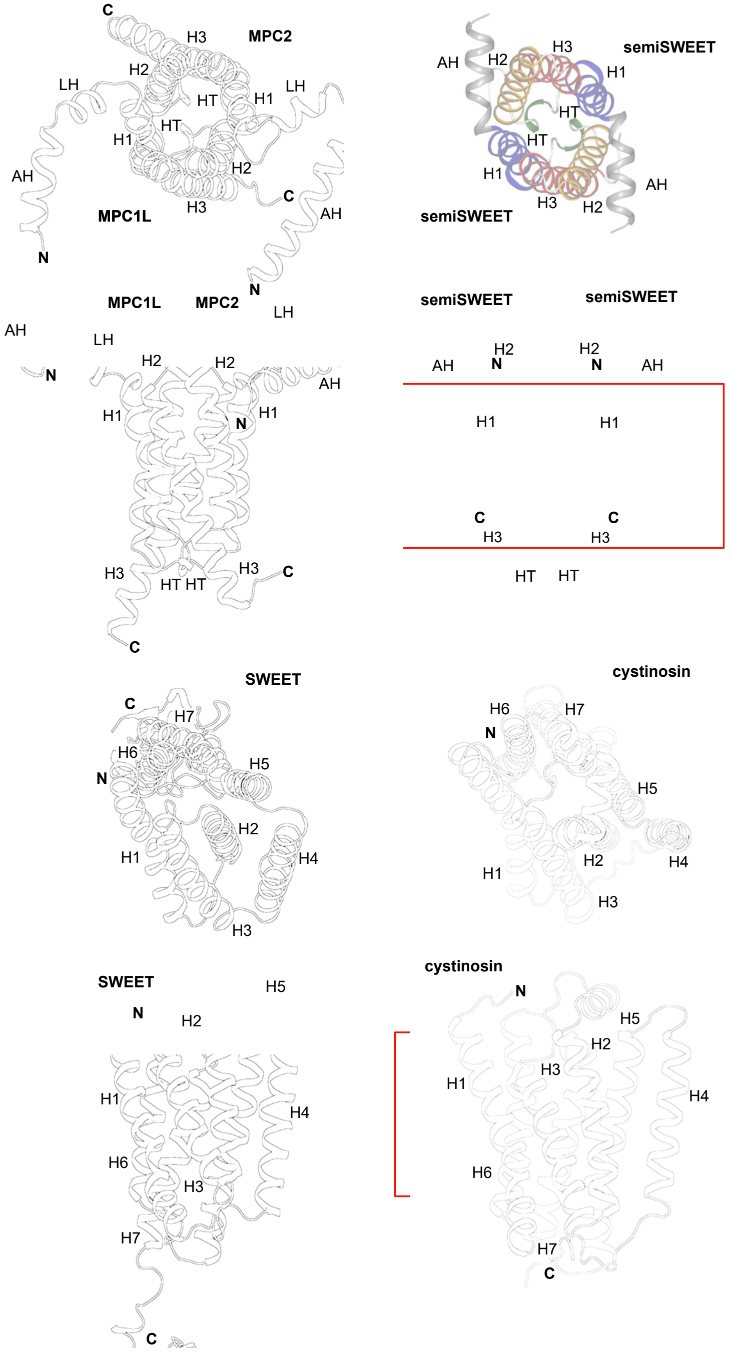

Fig. S24 Structural comparison of MPC to other members of the transporter-opsin-G protein-coupled receptor superfamily. Top and lateral views of the mitochondrial pyruvate

carrier (here), semiSWEET (4QND) (*47*), SWEET (5XPD) (*48*) and cystinosin transporter (7ZKW) (*49*). The color scheme of the helices is kept the same for comparison: amphipathic helices (AH) and linker helices are colored grey, transmembrane helix 1 (H1) is blue, helix 2 (H2) is yellow and helix 3 (H3) is red. The 3_{10} helix between H1 and H2 is in green. Lighter colors and labels are used for MPC2 or for transmembrane H4, H5, H6 of the SWEET and cystinosin transporters, where the extra helix linking the two halves is colored in magenta.

Table S1. Data collection, refinement and validation statistics.

	MPC-C7 (PDB: 9GIV, EMD: 51378)	MPC-mitoglitazone (PDB: 9GIY; EMD: 51381)	MPC-apo inward (PDB: 9GIX; EMD: 51380)
**Data collection and processing**			
Magnification	165,000	165,000	165,000
Voltage (kV)	300	300	300
Electron exposure ($e^-/Å^2$)	54.68	53.69	51.39
Defocus range (μm)	-1.8 to -0.6	-1.8 to -0.6	-1.8 to -0.6
Pixel Size (Å)	0.729	0.729	0.729
Symmetry imposed	C1	C1	C1
Initial particle images (no.)	1,682,367	1,555,900	1,329,798
Final particle images (no.)	199,754	190,059	164,876
Map resolution (Å)	3.65	3.79	3.65
FSC threshold	0.143	0.143	0.143
**Refinement**			
Model resolution (Å)	3.65	3.80	3.70
Initial model used	AF-Q9Y5U8-F1-model_v4_MPC1.pdb AF-O95563-F1-model_v4_MPC2.pdb	MPC-C7	MPC-C7
Map sharpening B factor ($Å^2$)	-105.3	-133.4	-114.0
**Model composition**			
Non-hydrogen atoms	2628	2795	2733
Protein residues	331	345	344
Ligands	1	1	0

B factors ($Å^2$) (min/max/mean)			
Protein	43.28/149.26/79.41	60.43/222.44/104.03	51.68/211.62/103.35
Ligand	74.60/79.79/76.75	81.82/90.30/86.61	
R.m.s. deviations			
Bond lengths (Å)	0.009	0.007	0.007
Bond angles (°)	1.480	1.059	1.101
Validation			
MolProbity score	0.86	1.10	0.78
Clashscore	1.35	3.09	0.92
Poor rotamers (%)	0.00	0.36	0.00
Ramachandran plot			
Favored (%)	99.08	99.12	99.11
Allowed (%)	0.92	0.88	0.89
Disallowed (%)	0.00	0.00	0.00

	MPC-zaprinast (PDB: 9GIW, EMD: 51379)
**Data collection and processing**	
Magnification	165,000
Voltage (kV)	300
Electron exposure ($e^-/Å^2$)	51.71
Defocus range (µm)	-1.8 to -0.6
Pixel Size (Å)	0.729
Symmetry imposed	C1
Initial particle images (no.)	781,930

Final particle images (no.)	295,345
Map resolution (Å)	3.92
FSC threshold	0.143
**Refinement**	
Model resolution (Å)	3.90
Initial model used	MPC-C7
Map sharpening B factor (Å^2)	-145.6
**Model composition**	
Non-hydrogen atoms	2661
Protein residues	333
Ligands	1
B factors (Å^2) (min/max/mean)	
Protein	61.94/295.18/147.53
Ligand	106.22/127.52/113.89
R.m.s. deviations	
Bond lengths (Å)	0.008
Bond angles (°)	1.346
Validation	
MolProbity score	0.86
Clashscore	1.33
Poor rotamers (%)	0.00
Ramachandran plot	
Favored (%)	98.17
Allowed (%)	1.83
Disallowed (%)	0.00

Table S2 Primers used for the generation of single alanine replacement mutants

Primer Name	Oligo Sequence
MPC1L_for	AAAAGAGCTCATGGCAAGAATGGCTGTTTATGGAGAAAAATGAGAGATA
MPC1L_rev	TTTTGAATTCTTATTATTATGATGCTTGTTTTGGTGGTTGTGAACCTGGA
MPC2_for	AAAGGATCCATGTCTGCTGCAGGTGCTAGAGGTTTGAGAG
MPC2_rev	TTTTTCTAGATTATCATTAGTGATGGTGATGATGGTGGTGATGTTCAGAAGTTGCGGTT
S35A_for	ATTTTTGGGGTCCAGCTTTTGCATGGGGTTTACCATTGGCTGC
S35A_rev	GCAGCCAATGGTAAACCCCATGCAAAAGCTGGACCCCAAAAAT
Y64A_for	GACTACAGCATTGATCTTGGCTTCTGCTATCTTTATGAG
Y64A_rev	CTCATAAAGATAGCAGAAGCCAAGATCAATGCTGTAGTC
F71A_for	CTCTGCTATCTTTATGAGAGCTGCATACAGAGTTCAACCAAG
F71A_rev	CTTGGTTGAACTCTGTATGCAGCTCTCATAAAGATAGCAGAG
T78A_for	GTGCCGTCTTAATGGCTGCTGGTTTTATCTGGTCAAGATA
T78A_rev	TATCTTGACCAGATAAAACCAGCAGCCATTAAGACGGCAC
L82A_for	TTCAACCAAGAAACTTGTTGGCGATGGCTTGTCATTGTACAAA
L82A_rev	TTTGTACAATGACAAGCCATCGCCAACAAGTTTCTTGGTTGAA
W82A_for	TAATGGCTACAGGTTTTATCGCTTCAAGATACTCATTGGTTAT
W82A_rev	ATAACCAATGAGTATCTTGAAGCGATAAAACCTGTAGCCATTA
L38A_for	GTCCAGCTTTTTCATGGGGTGCTCCATTGGCTGCATTCAAGG
L38A_rev	CCTTGAATGCAGCCAATGGAGCACCCCATGAAAAAGCTGGAC
F68A_for	CAGCATTGATCTTGTACTCTGCTATCGCTATGAGATTCGC
F68A_rev	GCGAATCTCATAGCGATAGCAGAGTACAAGATCAATGCTG
L96A_for	TTATCCCTAAAAATTGGTCTGCGTTCGCTGTTAACTTTTTCGT
L96A_rev	ACGAAAAAGTTAACAGCGAACGCAGACCAATTTTTAGGGATAA
K49A_for	TTTTCTGGGCACCAATAATGGCATGGGGTTTGGTTTGTGCTGG
K49A_rev	CCAGCACAAACCAAACCCCATGCCATTATTGGTGCCCAGAAAA

H86A_for	ACTTGTTGTTGATGGCTTGTGCTTGTACAAATGTTATGGCACA
H86A_rev	TGTGCCATAACATTTGTACAAGCACAAGCCATCAACAACAAGT
I89A_for	GGTCAAGATACTCATTGGTTGCTATCCCTAAAAATTGGTCTTT
I89A_rev	AAAGACCAATTTTTAGGGATAGCAACCAATGAGTATCTTGACC

Movie S1. Conformational changes of the human mitochondrial pyruvate carrier (MPC). Showing a morph between the outward-open (mitoglitazone-inhibited) and inward-open (apo) states of MPC. The conformational change is composed of two coupled movements for both subunits: a rigid-body motion of the H2-H3 bundle, and a movement of AH-LH-H1 about a hinge in H1.

Movie S2. Conformational changes of the human mitochondrial pyruvate carrier (MPC). Showing a sequence of conformations between the outward-open and inward-open states of MPC and back, based on AlphaFold 2 models (Fig. 2). Shown is a cross-section.
